# The Versatility in the Applications of Dithiocarbamates

**DOI:** 10.3390/ijms23031317

**Published:** 2022-01-24

**Authors:** Timothy O. Ajiboye, Titilope T. Ajiboye, Riadh Marzouki, Damian C. Onwudiwe

**Affiliations:** 1 Material Science Innovation and Modelling (MaSIM) Research Focus Area, Faculty of Natural and Agricultural Sciences, Mafikeng Campus, North-West University, Private Bag X2046, Mmabatho 2735, South Africa; 32480342@student.g.nwu.ac.za; 2 Department of Chemistry, Faculty of Natural and Agricultural Sciences, Mafikeng Campus, North-West University, Private Bag X2046, Mmabatho 2735, South Africa; 3 Food Security and Safety Niche Area, Faculty of Natural and Agricultural Sciences, Mafikeng Campus, North-West University, Private Bag X2046, Mmabatho 2735, South Africa; 37781294@student.g.nwu.ac.za; 4 Chemistry Department, College of Science, King Khalid University, Abha 61413, Saudi Arabia; riadh.marzouki@hotmail.fr; 5 Chemistry Department, Faculty of Sciences of Sfax, University of Sfax, Sfax 3029, Tunisia

**Keywords:** dithiocarbamate, metal complexes, medical use, industrial applications, agricultural applications

## Abstract

Dithiocarbamate ligands have the ability to form stable complexes with transition metals, and this chelating ability has been utilized in numerous applications. The complexes have also been used to synthesize other useful compounds. Here, the up-to-date applications of dithiocarbamate ligands and complexes are extensively discussed. Some of these are their use as enzyme inhibitor and treatment of HIV and other diseases. The application as anticancer, antimicrobial, medical imaging and anti-inflammatory agents is examined. Moreover, the application in the industry as vulcanization accelerator, froth flotation collector, antifouling, coatings, lubricant additives and sensors is discussed. The various ways in which they have been employed in synthesis of other compounds are highlighted. Finally, the agricultural uses and remediation of heavy metals via dithiocarbamate compounds are comprehensively discussed.

## 1. Introduction 

**Figure 1:**
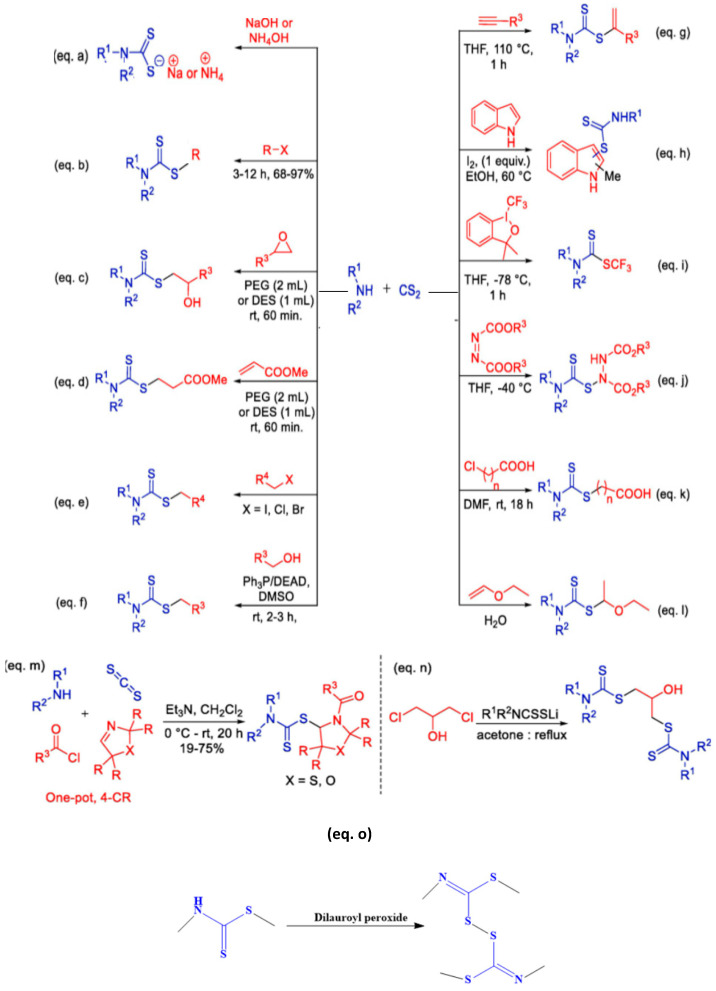
Various routes for the synthesis of dithiocarbamates. Adapted from [3]. Copyright (2020), with permission from Elsevier.

Dithiocarbamates are amides formed from dithiocarbamic acid and they have the ability to form stable metal complexes as a result of their exceptional coordination properties [1]. They could generally be classified as heterocyclic dithiocarbamates, symmetric dithiocarbamates, unsymmetric dithiocarbamate, dialkyldithiocarbamates and monoalkyldithiocarbamates [2]. Several methods have been used to synthesize dithiocarbamate compounds. However, the synthesis is commonly achieved by the reaction of carbon disulphide and amine (primary or secondary). The reaction is usually carried out in the presence of electrophiles such as imines, transition metals, epoxides and alkyl halides [3]. The synthesis could be effected without a catalyst or in the presence of an appropriate alkali as shown in Figure 1 through (equation a–n). Their ligands can form complexes with octahedral, square planar or tetrahedral geometry depending on the type of metal ion and also the ratio of the metal-to-ligand [2]. Dimers of dithiocarbamates are also formed by using dilauroyl peroxide as the oxidizing agent [4] (equation o). Other polyfunctional ligands of dithiocarbamate exist but they are rare compared to other forms of dithiocarbamate compounds [5]. Both the dithiocarbamate ligands and complexes are useful in several applications. However, when both ligands and complexes found relevance in similar applications, the complexes appear to be more potent than the ligands. For instance, dithiocarbamate complexes are more active against microbes than the ligands from which the complexes are formed [6]. The choice of dithiocarbamates compared to other related compounds is attributed to its poor solubility in water, ease of preparation under laboratory conditions, and formation of more stable compounds than several complexes made from other common analytical ligands [7].

**Figure 2:**
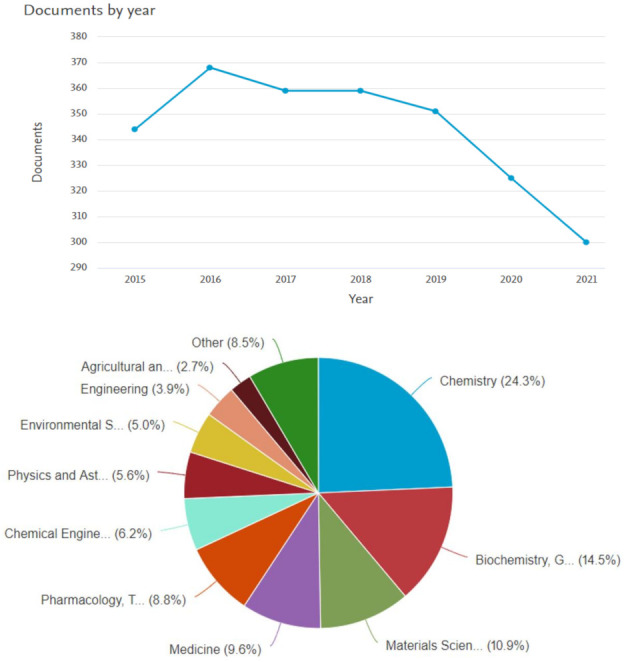
Statistics of publications on dithiocarbamate from 2015 to 2021 from Scopus database, accessed on 21 November 2021.

The study and discovery of different novel dithiocarbamate derivatives continues to increase as the different areas of their application are being investigated. As shown in the statistical data obtained from Scopus database (insert date), there are 2406 publications on dithiocarbamate from 2015, out of which 2264 (94.1%) are research articles (Figure 2). The publications on dithiocarbamate reached a peak in 2016, and within this range, chemistry researchers are at the forefront of the dithiocarbamate studies. Szolar reviewed the different ways of identifying and analyzing dithiocarbamates [8], while other reports focused only on some fragments of the applications. However, the need to review the comprehensive applications of the dithiocarbamate compounds is rife [9]. Consequently, this review gives an up-to-date and detailed account of various areas of applications of dithiocarbamate compounds including agriculture, medicine, industries, catalysis and in synthesis. These different areas of applications will be discussed in more detail in the following sections.

## 2. Heavy Metals Concentration and Remediation

Polluted samples usually consist of a mixture of organic (such as parabens, organochlorine pesticides and dyes) [10,11] and inorganic pollutants (such as heavy metals and nitrates) [12,13]. In some cases, there may be a need to remove one pollutant in the presence of other pollutants in the environmental samples. Several strategies have been used for concentrating heavy metals before their removal from the environmental samples. Both concentrating and removal of heavy metals from different media have been achieved through the use of dithiocarbamate compounds. Some of these dithiocarbamates as well as the heavy metals that were concentrated and removed are discussed in this section.

### 2.1. Heavy Metals’ Removal from the Environment through Dithiocarbamate Compounds

The ability of dithiocarbamate to selectively and strongly bind to most metal ions to form organometallic complex makes them a useful candidate for removing heavy metals from the environment [14,15]. The presence of two sulphurs with lone pairs of electrons makes it possible for dithiocarbamates to form chelate with these metals as well. However, it is possible for dithiocarbamate to use one of the sulphur donor atoms to form a bond with the metals. In short, it can act as bidentate or monodentate ligand [14]. Another factor that makes them particularly useful for metals with a variable oxidation state is their ability to stabilize these metals irrespective of their oxidation states and this can be explained by the oxygen bonding ability of the conjugates formed by dithiocarbamates [16,17]. The possibility of sharing electrons between the metal ions, sulphur atoms and nitrogen atoms coupled with the formation of metal complexes that cannot dissolve in water also makes them a better heavy metal chelator from the environmental samples [18].

As a result of these features, the use of dithiocarbamates to solve the problem of heavy metal pollution has been investigated and it was found to be a good metal chelator. In the studies conducted by Ayalew et al. [19], different amine-modified dithiocarbamates were used to successfully remove zinc, nickel and copper from wastewater at a low pH. The modified dithiocarbamate used for the investigation were tetraethylenepentamine-dithiocarbamate, triethylenetetramine-dithiocarbamate, diethylenetriamine-dithiocarbamate and ethylenediamine-dithiocarbamate. However, the three heavy metals were removed, but the amount of copper removed was more than the amount of zinc and nickel removed. Dithiocarbamates were also used to modify carbon compounds and then to remove heavy metals from wastewater. Trivalent arsenic has been removed in this way and dithiocarbamate was used to modify the cellulose that was used for trivalent arsenic removal [20]. Hydrochar is another carbon-based compound that was modified with dithiocarbamate and this also was found to be effective in removing divalent lead from the environment [21]. More than 90% of the heavy metals (lead(II), copper(II), and cadmium(II)) were removed from wastewater within 40 min when dithiocarbamate was grafted into crosslinked polymer made from glutaraldehyde and polyethyleneimine [22]. Other investigations involving the removal of heavy metals via dithiocarbamates are shown in Table 1.

The efficiency of heavy metals removal depends on the type of dithiocarbamate used for metal chelating. For instance, the metal chelating ability of diphenyldithiocarbamate ligands was found to be better than the chelating ability of diethyldithiocarbamate that did not contain a phenyl group [18]. Apart from the use of dithiocarbamates in the removal of heavy metals, they have also been used to determine and concentrate heavy metals instead of using surfactants [29,30,31].

### 2.2. Trace Elements Concentration and Determination through Dithiocarbamate Compounds

The determination of trace metals usually involves separation and pre-concentration stages. Dithiocarbamate compounds have been used for these purposes and this could be attributed to their selective and chelating properties. Activated carbon coated with phenylpiperazine dithiocarbamate was successfully used to concentrate Pb, Cd, Cu and Mn before they were determined by the flame atomic absorption spectrophotometry (FAAS) method [32]. Ammonium pyrrolidine dithiocarbamate, on glass fibre base, was also used to form a chelate complex with metal ions, which was followed by methyl isobutyl ketone elution and atomization of the metal ions. The quantification of the atomized sample was then carried out through high performance liquid chromatography (HPLC) [33]. When the multi-element determination of heavy metal ions was carried out through HPLC, dithiocarbamate was included in the column to improve the performance of the method [34]. Dithiocarbamate-modified silica gel was also employed for pre-concentration and separation of ions of several precious metals prior to their determination via inductively coupled plasma [35]. Table 2 shows other specific examples of investigations where dithiocarbamates were used to quantify metals.

## 3. Application of Dithiocarbamate Compounds as Stationary Phase in Chromatography

Dithiocarbamate compounds were also used as a component of the stationary phase during ligand exchange chromatography. They were useful for this application due to their strong chelating ability. Yeh and co-workers [42] utilized dithiocarbamate coated on silica as the stationary phase in the separation of heavy metals. It was observed that the amount of mercury taken up by this stationary phase was high, which could be attributed to the presence of extra complexing-nitrogen atoms from dithiocarbamate present in the stationary phase. In the chromatographic determination of multiple heavy metals, diethyldithiocarbamate and pyrrolidinedithiocarbamate were deposited on the Sep-Pak cartridge, which was used as the stationary phase. The method was able to determine these heavy metals even at μg l−1 level [43].

## 4. Application of Dithiocarbamate Compounds as Catalysts

Catalytic application of dithiocarbamate is another aspect that has attracted lots of research attention. It has been used for the synthesis of catalyst during organic synthesis as well as catalysts in polymerization. Some of these applications are explained in this section.

### 4.1. Application of Dithiocarbamate Compounds as Catalyst in Organic Transformation

**Figure 3:**
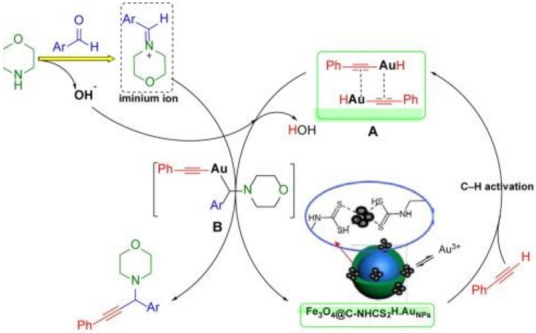
Mechanism for the dithiocarbamate-containing Au-catalyzed A^3^ coupling. Reprinted from [44]. Copyright (2021), with permission from Elsevier.

Core/shell nanostructures have been functionalized with magnetic dithiocarbamate deposited on gold and utilized as the catalysts for synthesizing propargyl amines through A^3^ coupling reaction [44]. The catalyst displayed good performance for the synthesis of propargyl amines when phenylacetylene, benzaldehyde and morpholine were used as the starting material. Further probe into the mechanism of the reaction showed that the reaction proceeded through a process involving the formation of iminium ion intermediate and C-H activation as shown in Figure 3. The choice of metal dithiocarbamate was as a result of its good solubility in organic solvents, chemical stability and the fact that it can be easily used in the anhydrous form [45,46].

The need to obtain carbon fibres with improved surface energy, roughness and chemical inertness led to the use of dithiocarbamate in its synthesis. Two of the methods that have been used with the incorporation of dithiocarbamates are Markovnikov addition and alkaline synthesis method. Guan et al. [47] utilized nickel dithiocarbamate compound as catalyst for enhancing the properties of carbon fibres by using both Markovnikov addition and alkaline synthesis. These carbon fibre are used as photopolymerization catalysts.

### 4.2. Application of Dithiocarbamate Compounds as RAFT Agent in Polymerization

Simultaneous control of stereoregularity and molecular weight of polymers is beneficial in polymer synthesis but it is difficult to achieve [48]. The use of RAFT (reversible addition–fragmentation chain transfer) agents has made simultaneous control feasible and different dithiocarbamate compounds have been investigated as RAFT agent [49]. Nitrogen-containing dithiocarbamates are now being used as the most effective RAFT agent with reduced bulky attachment when compared to other RAFT agents [50]. The presence of nitrogen in the dithiocarbamate compound stabilizes the cationic intermediate due to the fact that nitrogen is an electron-donating atom [48,50]. Dithiocarbamate was also used as both emulsifier and RAFT agent in the polymerization of stable latex of vinyl acetate polymer [51]. They are often used along with other RAFT agents for better control of tacticity and molecular weight. For instance, thiocarbonylthiol compound was included in the RAFT agent used for polymerization of vinyl ethers in the presence of Lewis acid catalysts [48].

## 5. Application of Dithiocarbamate in Synthesis

Dithiocarbamate compounds have been useful in the synthesis of organic intermediate as well as chalcogenides of metals. This section presents some of these synthesized compounds.

### 5.1. Application of Dithiocarbamate Compounds as Precursors in Material Synthesis

Different synthetic methods have been used to produce metal sulphide nanoparticles and one of these methods involves the use of metal complexes as single source precursors (SSP). Among the metal complexes used as SSP, dithiocarbamate complexes have being the most explored complexes. In our laboratory, we have synthesized some dithiocarbamate complexes, which were thermolyzed to generate metal sulphides [52]. Some of these nanoparticles (especially the bismuth based) have been reviewed by Ajiboye et al. [53]. The use of dithiocarbamate complexes for the synthesis of these nanoparticles is preferred since dithiocarbamate is rich in sulphur; hence, the use of a separate sulphur source will not be required [54]. Generally, the synthesis from the dithiocarbamate complex using the solvothermal method requires the use of capping agents such as oleylamine, octadecene, dodecane thiol, ethylene glycol and hexadecylamine. Their presence in the system controls the growth of the nanoparticles [55], while some of these capping agents (such as oleylamine) can also function as reducing agent, solvent or surfactant in the material synthesis [56]. Table 3 highlights other examples of nanoparticles made from dithiocarbamates.

### 5.2. Application of Dithiocarbamate Compounds in the Synthesis of Organic Intermediates

**Figure 4:**
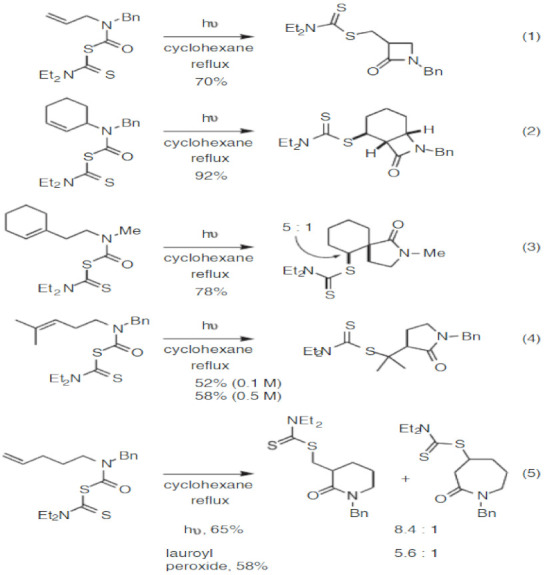
Synthesis of lactams (four-eight membered ring). Reprinted from [69]. Copyright (2007), with permission from Wiley and Sons.

The light-catalyzed reaction of dithiocarbamates in cyclohexane or chlorobenzene solvent leads to the formation of dithiocarbamate-containing lactam. The fact that the product contains dithiocarbamate makes it suitable for other dithiocarbamate-based applications [69]. Examples of lactam produced from dithiocarbamate are shown in Figure 4. Diethyldithiocarbamate has been used for the synthesis of ferrugine through a reaction that involves refluxing in the presence of cyclohexane and light [70].

**Figure 5:**
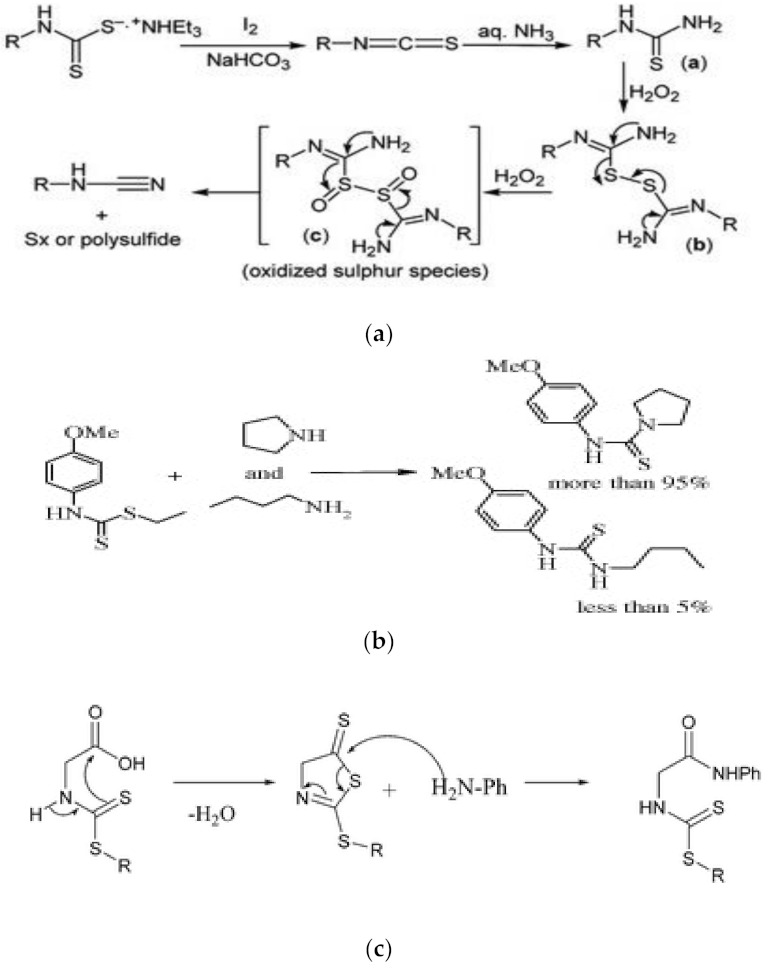
(**a**) Synthesis of cyanamide from dithiocarbamate. Reproduced from [71]. Copyright (2012), with permission from Taylor and Francis. (**b**) Synthesis of thiourea from dithiocarbamate and amines. Reproduced from [73]. Copyright (2009), with permission from Elsevier. (**c**) Synthesis of amide from dithiocarbamate. Reproduced from [75]. Copyright (2011), with permission from Royal Society of Chemistry.

The synthesis of cyanamide, which is an important intermediate for synthesizing pharmaceutical compounds, has been a serious challenge to researchers because its synthesis involves the use of highly toxic cyanogen halide. The synthesis is now carried out in a ‘greener’ way by using dithiocarbamate for its synthesis. Other reactants used for the synthesis are sodium bicarbonate, molecular iodine and hydrogen peroxide. The hydrogen peroxide functions as the oxidizing and desulphurizing agent. Other intermediates such as 1-phenylthiourea and isothiocyanates were formed during the synthesis [71]. The mechanism of the whole process is shown in Figure 5a. Another intermediate that was synthesized by using dithiocarbamate is thiourea, and synthesis via this procedure was preferred because toxic reagents such as hydrogen sulphide and thiophosgene were not needed [72]. Moreover, harsh reaction conditions such as the use of strong base or acid, elongated time of reaction and high temperature of the reaction are not required, unlike the other known synthetic routes [73]. In short, the synthesis is carried out by reacting dithiocarbamate with either ammonia, primary aliphatic or aromatic amine and a secondary aliphatic amine at 60 degrees Celsius. It could be carried out without using solvent or catalyst [73]. As shown in Figure 5b, thiazolidine-2-thiones synthesis has also been achieved from dithiocarbamate through a three-step method involving iodocyclization, dehydrohalogenation and nucleophilic substitution reactions [74]. The synthesis of novel amide was also feasible when dithiocarbamate compound was used as the starting material [75] as shown in Figure 5c.

Aryanasab and co-workers [76] reacted acid hydrazides with S-alkyl dithiocarbamates for synthesizing 1,3,4-thiadiazoles. The procedure was applauded because its cyclization step does not involve toxic catalysts or dangerous organic solvents. Apart from this specific reaction, it has general applicability. For instance, the reaction was used to prepare 2-amino-1,3,4-thiadiazoles by reacting acid hydrazides with dithiocarbamate.

## 6. Application of Dithiocarbamate Compounds in Agriculture

One of the uses of dithiocarbamate is in the eradication of diseases of plants and livestock. They have been used as pesticides to either prevent or eliminate plants’ diseases. The growth of unwanted plants has also been prevented or eliminated through the use of dithiocarbamate compounds. Some of the dithiocarbamate compounds that have been used for these applications are discussed in this section.

### 6.1. Application of Dithiocarbamate Compounds as Herbicides

**Figure 6:**
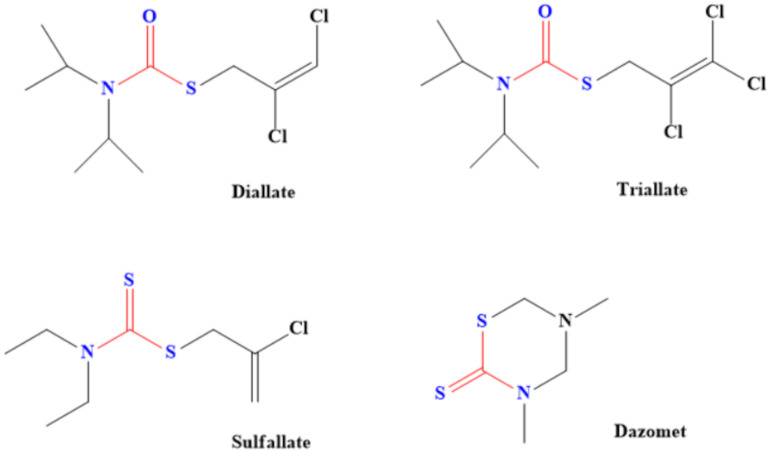
Examples of common dithiocarbamate-based herbicides. (One of the sulphur in dithiocarbamate has been replaced in diallate and triallate).

Dithiocarbamate-based herbicides contain groups such as dimethyldithiocarbamate, ethylenebis(dithiocarbamate) and propylenebis(dithiocarbamates). Examples of dithiocarbamate-containing herbicides are Metiram, Dazomet, Thiram, Disulfiram, Propineb, Maneb, Ziram and Zineb [77], although some of them are also used as pesticides. These herbicides are majorly used to prevent the growth of some broadleaf weeds as well as plants such as crabgrass, cheatgrass, bromegrass and foxtail [78]. Even plant that generates oxidants (active oxygen species) was successfully eliminated through dithiocarbamate herbicides [79]. Adjustment of the lipophilic and hydrophilic properties of dithiocarbamate by introducing groups such as sodium salts of dibutyldithiocarbamic acids, hexyl (2-(2- ethoxyethoxy) ethyl) dithiocarbamic acid, butyl (2-(2-ethoxyethoxy) ethyl) dithiocarbamic acid and ethyl (2-(2-ethoxyethoxy) ethyl)-dithiocarbamic acid was found to aid the action of dithiocarbamate as the pesticide. This is because of better penetration of plant cuticles compared to when ordinary sodium diethyldithiocarbamate was used as the herbicide [79]. Diallate, Sulfallate, Dazomet and Triallate are other common dithiocarbamate-based herbicides (Figure 6). Diallate [S-(2,3 dichloroallyl-)diisopropylthiocarbamate] is used to control monocotyledon weeds and it acts by attacking their fatty acids [80].

### 6.2. Application of Dithiocarbamate Compounds as Pesticides

**Figure 7:**
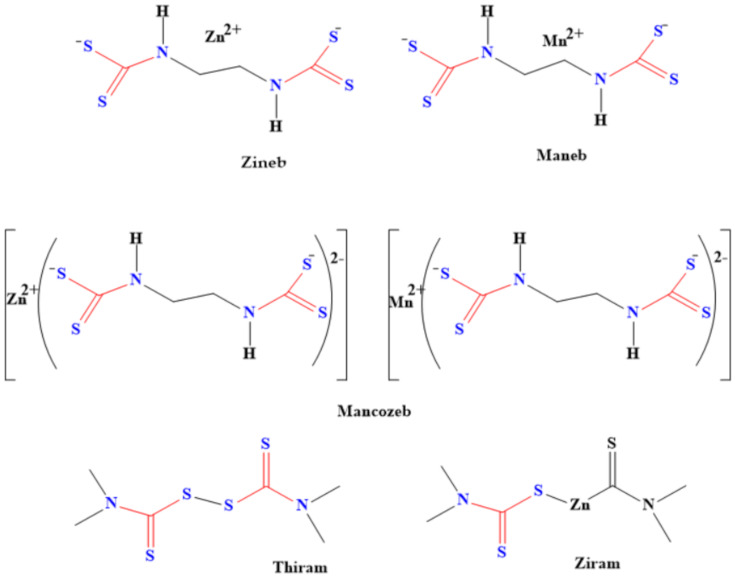
Examples of dithiocarbamate pesticides.

Pesticides made from dithiocarbamates are used as fungicides for various crops during processes such as shipment, storage and growth [81]. The structures of some of these dithiocarbamate-based pesticides are shown in Figure 7. These pesticides also kill the larva of some pests that cause plants’ and farm animals’ diseases, thereby boosting food security. For instance, both tricyclohexyltin and triphenyltin N-n-butyldithiocarbamate have been used as larvicide against the larva of *Aedes aegypti* and *Anopheles stephensi* mosquitoes [82]. These dithiocarbamates were found to be effective against the larva of these mosquito species. Moreover, Meloidogyne incognita, which is a disease caused by nematode, was eradicated by using dithiocarbamate derived from chitin oligosaccharide [83]. The derived dithiocarbamate pesticide has high activities for eliminating the nematode. In addition, it inhibits the hatching of eggs, thereby decreasing the population of the nematodes [83]. Specific examples of how these pesticides are being used are shown in Table 4.

## 7. Medical Applications of Dithiocarbamate Compounds

**Figure 8:**
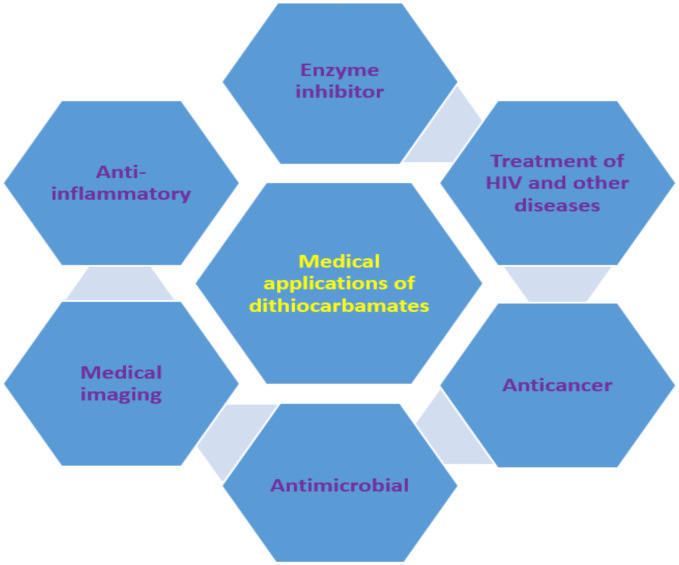
Medical applications of dithiocarbamate compounds.

The use of dithiocarbamate compounds in medicine has been investigated for more than 40 years [96]. One such application is their use as anti-angiogenic agent and they are usually evaluated for this application by studying their potential to heal wounds. For example, thalidomide dithiocarbamate was evaluated for wound healing to confirm its usage as the anti-angiogenic agent [97]. Dithiocarbamate ligands and complexes have also been studied for magnetic resonance imaging and other radiopharmaceutical imaging [96]. Gold nanoparticles functionalized with biomimetic amino acid dithiocarbamate were used as nanoprobe for cell imaging as a result of their negligible toxicity to human cells. This dithiocarbamate compound showed an enhancement factor of 9.8 × 10^5^ when used for surface-enhanced Raman scattering imaging [98]. Generally, the medical applications could be ascribed to their ability to form metal chelate and the high reactivity of dithiocarbamate anions to other moieties (such as thiol) [98,99]. Other medical applications of dithiocarbamate, which are discussed in this review, are summarized in Figure 8.

### 7.1. Application of Dithiocarbamate Compounds as Enzyme Inhibitor

A hydrolyzing enzyme (α-Glucosidase), which is important in the breaking down of starch and carbohydrate to glucose, is usually a target enzyme in the treatment of diabetes mellitus [100,101]. Among the compounds that has been used for the inhibition of this enzyme, coumarin-dithiocarbamate scaffold has proven to be very effective and this has made it a useful compound in the treatment of type 2 diabetes. Coumarin-dithiocarbamate is a competitive inhibitor of α-glucosidase since it binds to its active site as evidenced by results obtained from molecular docking [101]. Specifically, there is formation of a hydrogen bond between the amino acid (His279) and coumarin moiety [100]. Pyrrolidine dithiocarbamate has also been used as an effective inhibitor of enzymes, specifically the nuclear factor kappa B [102]. A metalloenzyme, carbonic anhydrase, which is involved in the reversible reaction of forming bicarbonate from carbon dioxide in the body has also been inhibited by the derivative of dithiocarbamate-sulfonamide [103]. Its inhibition is usually required when it starts to display abnormal activities in the body of animals, which may lead to physiological disorder such as altitude sickness, epilepsy, glaucoma, cerebral and retina oedema [104]. The treatment of ‘superbug’ infection has been made possible through the inhibition of metallo-β-lactamases which are responsible for the infection. Dithiocarbamates play significant roles in the inhibition of this enzyme because the carbonyls and hydroxyl group in some dithiocarbamate compounds effectively bind to the zinc in the active site of this enzyme, leading to their inhibition [105]. Dithiocarbamate coupled with phthalimide is a competitive inhibitor of butyrylcholinesterase and acetylcholinesterase. This inhibitive property makes it suitable for the treatment of Alzheimer’s Disease [106].

### 7.2. Application of Dithiocarbamate Compounds in HIV Treatment

Elimination of HIV is very challenging with the current retroviral treatment due to numerous latently infected CD4T cells. This is because the available treatment requires placing the patients on drugs for a long period of time and some of these drugs are associated with known side effects. However, the treatment is active in prolonging the survival of the patient, thereby reducing the mortality associated with HIV infections and minimizing the transmission of the disease [107]. The quest for the improvement of these existing medications has resulted in a continuous search for novel HIV inhibitors. Dithiocarbamate has been investigated as a possible HIV inhibitor. For instance, zinc-dithiocarbamate-*S*,S′-dioxidcyclic zinc-dithiocarbamate-*S*,S′-dioxide was used to effectively inhibit HIV. Specifically, HIV-1 was inhibited by mediating a cell-to-cell fusion between anti-CXCR4 and CXCR4 that is present on the cell’s surface [108]. Dithiocarbamate compounds have also been used to delay the progression of HIV into AIDS. Diethyldithiocarbamate has been reported for this purpose and it was found to be active in delaying the proliferation of the virus [109]. Pyrrolidine dithiocarbamate has also been found to be useful in the treatment of HIV because it inhibits the nuclear factor-κB [110]. It acts as an antioxidant to block the activation of HIV-1 and nuclear factor KB (NF-KB) since oxygen radicals play significant roles in the activation of HIV-1 and NF-KB [111]. Other studies have also established the link between nuclear factor-κB, immune systems and HIV [2,112]. Lang et al. [113] observed that the HIV symptoms were relieved, the immune function was enhanced overall and the progression of HIV was retarded when sodium dithiocarbamate was used as an oral drug for HIV patients. One of the reasons why sodium dithiocarbamate was found to be useful for this purpose was because of its relatively low toxicity when compared to other dithiocarbamates and this was further shown by its lethal dose (LD_50_), which was measured to be 1.5 g/Kg of body weight from the investigation conducted with rats and mice [114]. Furthermore, sodium dithiocarbamate drugs did not in any way initiate any major biological or clinical side effects [115].

### 7.3. Application of Dithiocarbamate Compounds in the Treatment of Other Diseases

**Figure 9:**
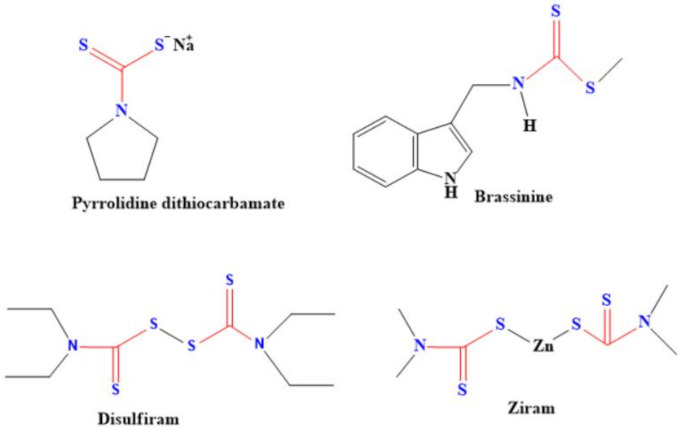
Representative of dithiocarbamate compounds used for the treatment of diseases.

Dithiocarbamates are also useful antiglaucoma agents, even better than sulfonamide dorzolamide which is a clinically-recognized drug for treating glaucoma [116]. That dithiocarbamate compounds are easy to prepare, coupled with their ability to lower the intraocular pressure, made them the preferred compounds compared to the sulfonamides [116]. The fact that dithiocarbamate compounds could inhibit carbonic anhydrase make them more suitable for treating glaucoma [117]. They can also inhibit carbonic anhydrase, which leads to the treatment of several diseases/disease conditions. Some of these diseases are edema, epilepsy, obesity, hypoxic tumor, inflammatory diseases, neuropathic pain, Alzheimer diseases and cerebral ischemia [117]. Pyrrolidine dithiocarbamate was reported for the repair of damaged lungs (lung edema) instead of lung transplant. Pyrrolidine dithiocarbamate acted by inhibiting NF-Κb, thereby suppressing the activation of immunity during lung reconditioning via ex vivo lung perfusion [118]. In addition to lung treatment, this dithiocarbamate compound was used for the treatment of epilepsy by protecting the piriform cortex of the cerebrium without causing loss of hilar neuronal [119]. There was an increase in the generation of reactive oxygen species in the renal cortical and a decrease in the lipoprotein level of the tested animals that were fed with water containing pyrrolidine dithiocarbamate [120]. In fact, some dithiocarbamate compounds are under clinical trial for the treatment of corona virus (SARS-CoV-2) [9]. Examples of the common dithiocarbamates used for the treatment of various diseases are shown in Figure 9.

Dithiocarbamate drugs (Propineb, Zineb and Maneb) were also found to be useful in the treatment of leishmaniasis, a protozoan disease [121]. The disease is common in the subtropical and tropical countries and has claimed several lives [122]. Before the discovery of dithiocarbamate drugs, miltefosine, paromomycin and amphotericin, which are expensive, were used but it was discovered that the disease had developed resistance against these drugs and some side effects were also reported [123]. The dithiocarbamate-based drugs were found to be particularly useful because they have no significant effect on the mammalian cells as they lead to the death of *Leishmania* cell with a lethal dose of 50% [121]. Bromine-containing ethylsarcosinedithiocarbamate of gold complex has been used to treat trypanosomiasis caused by *Trypanosoma brucei rhodesiense* and other parasites [124]. Apart from the fact that gold itself has inhibitory properties against these parasites, the amine-end of the dithiocarbamate compound also initiates the generation of reactive oxygen species leading to the death of the parasites [125]. Brassinin, which is a dithiocarbamate compound, and its derivatives have been found to be active against *Trypanosoma cruzi* (trypanosome that cause Chagas diseases). It has displayed a good antiproliferative effects that is similar to benznidazole and nifurtimox, which are well known antichagasic agents [126]. Apart from brassinin, Ochoa et al. [127] synthesized 34 dithiocarbamate compounds (3,5-disubstituted-tetrahydro-2H-1,3,5-thiadiazine-2-thione derivatives). Some of these compounds were reported for the treatment of Chagas diseases. They have the ability to generate reactive oxygen species, leading to oxidative damage of *Trypanosoma cruzi*. One of the psychological implications of diabetes 1 and 2 is anxiety [128]. Studies conducted by using mice showed that pyrrolidine dithiocarbamate showed anxiolytic-like effects [129]. Table 5 presents some of the diseases in which the use of dithiocarbamate compounds have found relevance.

### 7.4. Anti-Inflammatory Application of Dithiocarbamate Compounds

Aspirin and the non-steroidal anti-inflammatory drugs have side effects [141]. As a result of the side effects, alternative anti-inflammatory drugs that have minimal toxicity and side effects without compromizing the efficiency are required [142]. The dithiocarbamate-based compounds have also been discovered to possess anti-inflammatory properties. Song et al. reported the replacement of nitrogen position of indoles with dithiocarbamate groups at room temperature, which were found to inhibit the release of interleukin-6 and tumor necrosis factor alpha, thereby displaying anti-inflammatory properties [143]. This anti-inflammatory property was found to be useful in the treatment of acute lung injury because it perpetuates, amplifies and mediates anti-inflammatory injury, thereby leading to inflammatory response blockage [144,145]. Another dithiocarbamate compound that has been utilized for anti-inflammatory properties is pyrrolidine dithiocarbamate and one of the reasons why it is considered for this application is its stability at physiological pH in solution, in addition to its ability to traverse the cell membrane [146]. Pyrrolidine dithiocarbamate was effective against chronic and acute inflammation [147].

### 7.5. Anticancer Application of Dithiocarbamate Compounds

There are more than 10 million cases of cancer every year around the world [148], and it is one of the leading causes of death [149]. Hence, there is need for the synthesis of novel anticancer agents to complement the existing anticancer drugs. Several compounds containing dithiocarbamate have been investigated as anticancer agents and they act by inhibiting enzymes responsible for cancer growth (such as catalase), alter the production of reactive oxygen species or trigger the induction of apoptosis at the mitochondria [150]. For example, the ability of diethyldithiocarbamate to chelate copper was utilized in treating both breast and prostate cancer. This chemotherapeutic cancer treatment worked through the accumulation of copper in the cancerous tissues and cells [109]. The copper complexes also have the ability to initiate the inhibition of proteasome and cause apoptosis in the cancer cells of humans. Similar to copper dithiocarbamate, zinc dithiocarbamate was also found to have a similar effect on cancer cells but it occurs through a different mechanism. Despite the difference in mechanism, caplain is involved in the apoptotic cell death process of dithiocarbamate of both zinc and copper [151]. Dithiocarbamate complexes of trivalent gold have also been found to be effective against cancer cells [152]. Similarly, derivatives of benzoxazole with dithiocarbamate moieties were found to be active in the treatment of breast cancer [153]. Gamma glutamyl transferase was used as trigger for copper diethyldithiocarbamate prodrug and it was used for the treatment of prostate cancer, which is the second most common cancer among men. The drug showed high antiproliferative efficiency within 24 h in prostate cancer cells [154]. Thiocarbonylthiol compounds have been found to be a good anticancer agent with reduced toxicity when compared to cisplatin, a very known anticancer agent. The anticancer activity of thiocarbonylthiol occurs by inducing apoptosis and induction of DNA damage [155]. The anticancer activities of dithiocarbamate often occur via unrestrained cell death as a result of inflammation, hypoxia or other external damage leading to the release of the content of the cytoplasm into the surroundings. This cell damage through these means is termed necrosis [156].

### 7.6. Antimicrobial Applications of Dithiocarbamate

The presence of donor atom (sulphur) in dithiocarbamate compounds makes them possess good antimicrobial properties. So, they are able to form a chelate with positively charged metal ions. The sulphur atom reduces the polarity of the binding metal through the delocalization of electrons over the entire chelate ring. This process makes the permeability of the microbes feasible [157,158]. In some cases, there is formation of a hydrogen bond between the active center of the microbe and the –N_ (S)SH group of the dithiocarbamate, leading to an interference of the physiological processes of the cells [157,158]. The common micro-organisms that dithiocarbamate compounds have been used against are bacteria, fungi and virus. Different dithiocarbamate compounds that have acted against these microbes will be discussed in this section.

#### 7.6.1. Antibacterial Application of Dithiocarbamate Compounds

There is increased interest in the development of novel antibacterial substances as a result of the increase in the number of drug-resistant bacteria. Silver(I) dithiocarbamate triphenylphosphine complexes have showed better antibacterial properties, greater than ciprofloxacin against Gram (−) and Gram (+) bacteria. The bacteria used for the investigations are *Staphylococcus aureus*, *Salmonella typhimurium*, *Escherichia coli*, *Klebsiella pneumoniae* and *Pseudomonas aeruginosa.* It was observed that this dithiocarbamate complex displayed better antibacterial activity against Gram (+) positive bacteria than the Gram (−) negative bacteria with the exemption of *K. pneumonia.* The reduced activity of the dithiocarbamate complex could be linked to the fact that the cell wall of Gram (−) is made of several layers unlike the cell wall of Gram (+) bacteria, which is made of a single layer. Hence, the penetration of dithiocarbamate is hampered by multiple cell walls [159]. Another novel compound, 1,2,3-triazole-dithiocarbamate-naphthalimides, showed good antibacterial activity against *Staphylococcus aureus, Bacillus subtilis, Escherichia coli* and *Candida albicans.* Notably, this novel compound showed a better antibacterial performance than a common antibacterial drug (Cefuroxim) when it was tested against *B. subtilis* [160]. Derivatives of isatin dithiocarbamate have also been investigated as an antibacterial agent against both Gram (+) bacteria (*Strep. Pneumonia* and Staph*. aureas*) and Gram (−) bacteria (*Pseud aeruginosa* and *Escherichia coli*). It also showed satisfactory antibacterial activities against these bacteria when compared to antibacterial activities of some common drugs [161]. In all the antimicrobial investigations, the methods used were broth dilution, disc diffusion, zebrafish model, well diffusion, tube diffusion, agar dilution, broth micro-dilution methods or the combination of the methods. Other dithiocarbamate investigated for antimicrobial activities are shown in Table 6.

#### 7.6.2. Antifungal Application of Dithiocarbamate Compounds

The reduction in the plant yield as a result of fungal infections coupled with the negative impacts of fungi on the health of plants and animals makes the synthesis of efficient antifungal compounds of utmost priority. Dithiocarbamates are one of the numerous compounds that have been investigated as antifungal drugs. For example, two fungi (*Candida albicans* and *Candida tropicalis*) extracted from HIV patients that are also suffering from oral candidiasis were rendered passive in the presence of organotin(IV) dithiocarbamates. The organotin dithiocarbamate was able to achieve this by suppressing the ergosterol synthesis without cytochrome deactivation [168]. Plant pathogenic fungi have also been eradicated by using dithiocarbamate compounds as antifungal agents [169]. *Alternaria porri* and *Fusarium oxysporum,* which are plant pathogens were inhibited by using ammonium dithiocarbamate coupled with chitosan [170]. The inhibitory effect of this dithiocarbamate compound was clearly better than when chitosan alone was used as the antifungal agent. From the investigation conducted by Ferreira et al. [171] dithiocarbamate complexes containing nickel, platinum and palladium were found to be effective against several fungi (*Penicillium citrinum*, *Aspergillus niger*, *Aspergillus flavus* and *Aspergillus parasiticus*). The antifungal activities of these dithiocarbamates were found to be better than some known antifungal drugs (nystatin and miconazole nitrate). When the antifungal activities of dithiocarbamate complexes of nickel and palladium were compared by this same group [171], the nickel complexes were more effective against *Aspergillus parasiticus*, whereas palladium complexes were more effective against *Aspergillus flavus.* Three organotin dithiocarbamate compounds (tributyltin dithiocarbamate propionates, tributyltin dithiocarbamates and dibutyltin dithiocarbamates) have been reported to possess antifungal activities against fungi that destroy woods (*Coriolus versicolor*, *Coniophora puteana* and *Serpula lacrymans*). The antifungal activities of these dithiocarbamate compounds is comparable with that of tris-(benzyltriazolylmethyl)amine, a common antifungal compound [172].

#### 7.6.3. Antiviral Application of Dithiocarbamate Compounds

The treatment of several viral infections have been carried out via dithiocarbamate-containing ligands and complexes. One of the common dithiocarbamates that has been utilized for this purpose is pyrrolidine dithiocarbamate. It was used to alter the pathogenesis of cells infected with dengue virus and its high replication ability was inhibited. In fact, this dithiocarbamate was observed to be more active against dengue virus than gefitinib, which is a receptor inhibitor [173]. Enterovirus 71, which is a viral disease that affects the mouth, foot and hand of animals have been treated with pyrrolidine dithiocarbamate. There was significant reduction in the yield of the virus after cell culture was treated with this dithiocarbamate [174]. Antiviral properties of pyrrolidine dithiocarbamate have also been investigated against herpes simplex virus, influenza virus, rhinovirus and cox sackie virus B3 [174].

### 7.7. Application of Dithiocarbamate in Medical Imaging

**Figure 10:**
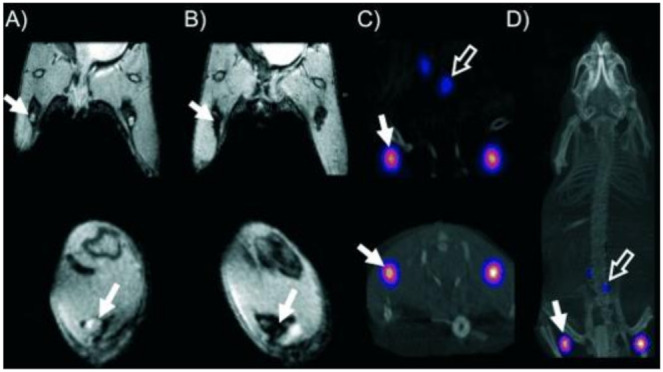
In-vivo dual MRI-PET images obtained from mouse using isotopic-labelled copper dithiocarbamate complex. (**A**,**B**) Popliteal nodes of coronal (**top**) and short axis (**bottom**) MR images of the lower abdominal area and upper hind legs before (**A**) and after (**B**) injecting dithiocarbamate imaging agents. (**C**) Coronal (**top**) and short-axis (**bottom**) images showing the uptake of the dithiocarbamate (**D**) image of the whole body of the mouse. Reprinted from [176]. Copyright (2011), with permission from Wiley and Sons.

Two dithiocarbamate ligands, (methoxyisobutyl dithiocarbamate) and tert-butyl dithiocarbamate, were radiolabeled with ^99m^Tc-nitrido core and used for myocardial imaging. These dithiocarbamate ligands performed better than ^99m^TcN(NOEt)_2_, which was already on phase III clinical trial for the same imaging application [175]. The synergistic application of magnetic resonance imaging (MRI) and positron emission tomography (PET) was achieved with radio-labelled copper dithiocarbamate bonded to iron trioxide. This dual modality imaging (Figure 10) was possible due to the accumulation of this dithiocarbamate compound in the lymph nodes without translocation of radioactivity to other parts of the tissues. The results were also obtained faster with less dose of radiation required compared to other common dual MRI-PET agents [176].

Ciprofloxacin dithiocarbamate has been radiolabeled with ^99m^TcN complex and used for imaging infections in mice. The binding affinity of the complex significantly improved compared with similar compounds without dithiocarbamate. Moreover, the complex was stable for more than 6 h at room temperature [177]. Dithiocarbamate compounds have also been useful in imaging tumor tissues and this is as a result of the good tumour/muscle ratios of these compounds. In addition, their high tumour uptake leading to their accumulation in the site containing tumors makes them suitable for imaging applications. An example of such a compound is ^99m^Tc(V)-glucoheptonate radiolabeled deoxyglucose dithiocarbamate [178].

## 8. Application of Dithiocarbamate Compounds in the Industries

**Figure 11:**
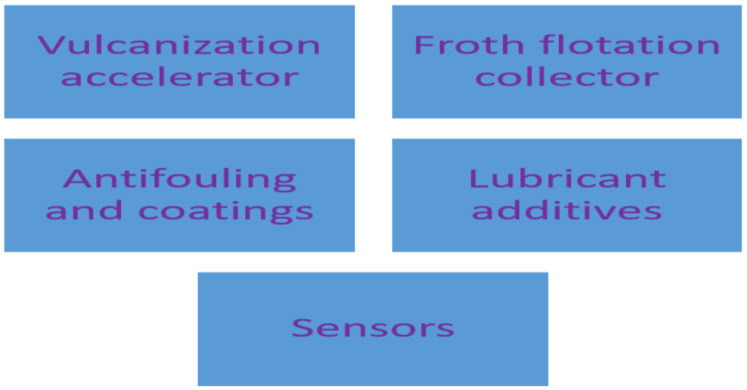
Industrial applications of dithiocarbamates.

Several industries are using dithiocarbamate as the starting materials in different industrial processes and this has spiked the consumption of dithiocarbamate compounds. Some of the industrial uses of dithiocarbamate compounds that will be discussed in this section are shown in Figure 11.

### 8.1. Application of Dithiocarbamate Compounds as Vulcanization Accelerator

Vulcanization accelerator is required for large scale production of rubber becaused it improves the state and rate of crosslinking of rubber during the process [179]. Thiocarbanilide, guanidine and aniline have been used as accelerators, and the vulcanization process (in their presence) was found to be faster than sulphur vulcanization. However, these accelerators showed different levels of toxicity [179,180]. Vulcanization of nitrile butadiene and other types of rubber are now speeded up by dithiocarbamate. This is due to its ability to simultaneously enhance the state and rate of vulcanization [179]. Wang et al. [181] investigated the effect of using sodium, zinc and lanthanium dithiocarbamate as a vulcanization accelerator. The vulcanization carried out with these dithiocarbamates was fast compared to the investigation without dithiocarbamate. Among the metal dithiocarbamates used for the investigation, lanthanium diethyldithiocarbamate was observed to perform better in accelerating vulcanization process. The rate of rubber acceleration further increased when rubber black was also added as an additive to assist dithiocarbamates. The carbon black has functional groups such as lactones and phenolic, which allows it to react with sulphur to form a network during vulcanization [182]. In a similar investigation, samarium lysine dithiocarbamate was reported to accelerate the vulcanization process and also boosted the crosslink of the network. The introduction of stearic acid and zinc oxide as the activators further enhanced the properties of the rubber produced [183].

Amine-containing zinc dithiocarbamates were also found to be effective as a vulcanization accelerator. Some of these dithiocarbamates are zinc (*N*-ethyl piperazino) dithiocarbamate and zinc (*N*-benzyl piperazino) dithiocarbamate. They were found to be safer and were able to improve the ability of rubber to withstand aging unlike zinc dimethyl dithiocarbamate [184]. Apart from using dithiocarbamate directly for accelerating vulcanization, the dithiocarbamates have also been found to be good precursors for preparing other materials that were used as the vulcanization accelerator. For instance, molybdenum dialkyl dithiocarbamate was used as a precursor for preparing molybdenium sulphide nanoparticles, which were then used as catalysts for speeding up the rate of vulcanization [185]. Sometimes, dithiocarbamates could be useful as a bridge for other structures, thereby resulting in a composite with improved vulcanization kinetics. This was demonstrated by using lanthanum glutamic dithiocarbamate to bridge silica with styrene butadiene rubber and the resulting composite was used as a vulcanization accelerator. Other examples of dithiocarbamates that were used as vulcanization accelerator are zinc diisononyldithiocarbamate, zinc isobutyldithiocarbamate, zinc dibenzyldithiocarbamate, zinc dibutyldithiocarbamate and zinc diethyldithiocarbamate [186]. Several modifications have been carried out to improve the performance of these dithiocarbamates as vulcanization accelerator. One such attempt is the use of zinc salts of butyl, isopropyl and ethyl xanthates along with these dithiocarbamates and it has yielded a positive outcome [182]. The introduction of phosphorus into dithiocarbamate to form phosphorylated dithiocarbamates has also been reported as a vulcanization accelerator and this was also discovered to give more positive results compared with ordinary dithiocarbamates [187].

The studies carried out by Nieuwenhuizen et al. [188] showed the use of zinc dithiocarbamate as a vulcanization accelerator. The complex acts as a mediator between the rubber and sulphur. It brings the sulphur atom in the ring of zinc dithiocarbamate and introduces it into the carbon–hydrogen bond through a reaction involving a double bond. The resultant product of this reaction is polythiothiol and some of them further undergo methathesis reaction leading to the formation of polysulphide. Desulfhydration of polythiothiols may also occur, leading to the formation of hydrogen peroxide and sulphides. These reactions and products lead to the increase in the speed of vulcanization.

### 8.2. Application of Dithiocarbamate Compounds as Froth Flotation Collector

In froth flotation, a collector is needed to capture the mineral that is needed. The principle upon which the collector acts is that the active sites of the mineral interact with the polar region of the collector, while the non-polar region of the collector binds to the bubbles. The combination of adsorbed mineral particles and the collector binds to the surface of the slurry, leading to efficient separation [189]. Several mineral (such as sulphides of lead, zinc and tin) ores have been obtained via the use of collectors as the flotation agent. Xanthates are common collectors that are used for this purpose, but it has been discovered that oxidized mineral ores showed insufficient response to xanthate collector [190]. This slow response necessitated the sulphidation of the oxidized minerals prior to conditioning with the collector so as to improve the performance of the process [191]. The sulphidations are carried out by using ammonium sulphide, sodium hydrosulphide or sodium sulphide [192]. To carry out flotation without sulphidation, hydroxamic acids were used as froth flotation collectors but their performance depends on the nature of the ore [192]. So, there is a need for a more efficient flotation collector.

Dithiocarbamate compounds have been investigated as a possible replacement for these known collectors. For example, 2-hydroxyethyl dibutyldithiocarbamate has been used as surfactant collector for the removal of galena from sphalerite. This was achieved by using 4 × 10^−4^ mol·L^−1^ of the dithiocarbamate compound. Its effectiveness was proven through the adsorption mechanism, which revealed that the presence of this dithiocarbamate improved the hydrophobicity of the surface of galena via the process of chemisorption. S-benzoyl-*N*,*N*-diethyldithiocarbamate is another flotation surfactant collector and its performance was better than that of isobutyl xanthate and diethyldithiocarbamate. Similar to 2-hydroxyethyl dibutyldithiocarbamate, S-benzoyl-*N*,*N*-diethyldithiocarbamate also displayed enhanced selectivity for galena in the presence of aphalerite [193]. In some cases, dithiocarbamates are used as co-collector along with other known collectors. Ngobeni et al. used both xanthates and sodium di-methyl-dithiocarbamate to separate nickel ores from pentlandite in a South African mine. Their study showed an enhanced nickel recovery when these co-collectors were used together. This indicated that selectivity of the collector improved in the presence of dithiocarbamate [194]. In another investigation, varied ratios of di-n-propyl dithiocarbamates and cyclo-hexyl dithiocarbamates were used as collectors along with other sulphur-containing collectors. The presence of dithiocarbamate resulted in the recovery of more than 80% of the ore. Finally, the recovery of galena from the ore containing several metallic sulphides was enhanced when S-benzyl-*N*-ethoxycarbonyl thiocarbamate was used as the collector. Its performance was even better than that of ammonium dibutyl dithiophosphate and sodium diethyl dithiocarbamate which are conventional collectors [68]. The same performance was observed when N-[(3-hydroxyamino)-propoxy]-*N*-octyl dithiocarbamate was used as the collector for extracting cassiterite [195].

Dithiocarbamate compounds also found application in the extraction of precious metals from their ores. This is connected to their usefulness as froth flotation collectors. S-cyanoethyl *N*,*N*-diethyl dithiocarbamate and S-cyanoethyl *N*,*N*-diethyl dithiocarbamate are two dithiocarbamate compounds that have the ability to form an undegraded compound with gold when it is in aqueous form. This ability qualifies them as collector for recovering gold from their ores. In addition, S-cyanoethyl *N*,*N*-diethyl dithiocarbamate also enhances the floatability of chalcopyrite, which makes it useful in the extraction of high quality copper with minimal arsenic contaminant [196]. Modified dibutyldithiocarbamate and diethyldithiocarbamate performed the same function in the extraction of gold from its ore with a better gold recovery [197].

### 8.3. Application of Dithiocarbamate Compounds as Antifouling/Electroplating Agents

The control of organisms responsible for fouling in the marine environment has been a subject of research, which has led to the use of dichlorodiphenyltrichloroethane/tributyltin, 8-methyl-N-vanillyl-6-nonenamide and triphenylborane pyridine as antifouling agents. Further research has shown that zinc ethylene(bis) dithiocarbamate can also perform a similar function [198]. Zinc dithiocarbamate was added to some known antifouling agents and the overall effect was discovered to be synergistic, which implies that the dithiocarbamate could be used alone or in a mixed form as antifouling agent [199]. Zwitterionic phenyl phosphorylcholine dithiocarbamate was able to lower the adsorption of protein into the surface of the gold electrode, thereby reducing fouling in these electrodes. The dithiocarbamate-containing zwitterionic phenyl phosphorycholine performed better than when diazonium salt was used to replace dithiocarbamate in the same compound [200].

### 8.4. Application of Dithiocarbamate Compounds in Coatings

The formation of coatings that is rich in phosphophyllite is possible when phosphate is being used for coating with the addition of long-chain dithiocarbamates. The effect is the rise in the soluble iron, wet adhesion and alkaline stability of the phosphate coatings. In short, the presence of dithiocarbamate as the additive makes electrophoretic deposition feasible [201]. The corrosion resistance and porosity of zinc-phosphated steel was also enhanced when dithiocarbamate compounds such as octadecyldithiocarbamate, hexadecyldithiocarbamate and dodecyldithiocarbamate were used as additives during the coating process [202]. The need to minimize acid mine drainage or acid rock drainage, which cause problems in the environment, led to the coating of the pyrite [203]. Some of the chemical species that have been used for this purposes are oxalic acid, natural lignin, fatty acid, humic acid and acetyl acetone and they all act by slowing down the oxidation of pyrite. However, their usage requires the use of hydrogen peroxide which also has a negative impact on the environment. Besides, coating involving phosphate and silicate has little stability when the pH is too low [204]. To overcome these challenges, sodium triethylenetetramine-bisdithiocarbamate has been used to coat pyrite. It acts by forming a passivating cross-link on the surface of the pyrite and the formed crosslink is not only hydrophobic but it also prevents the release of metals even at a low pH [204].

### 8.5. Application of Dithiocarbamate Compounds as Lubricant Additives

**Figure 12:**
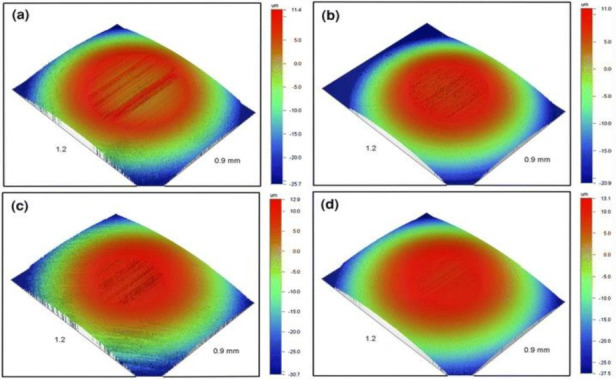
(**a**) The lubricating performance of oil without dithiocarbamate additives compared with the oil incorporated with dithiocarbamates (**b**) DBB-EBzDTC (**c**); DOB-EBzDTC and (**d**) DOB-EEDTC. Reprinted with permission from Springer Nature, Tribology letters [210]. Copyright (2011).

Improvement of lubricants is vital for the durability and efficiency of energy generated in the machines [205]. One of the strategies adopted to enhance the quality of lubricants is to introduce additives. Other reasons for introducing additives to lubricants are to cut down the gas environmental pollutants and to minimize the consumption of fuels [206]. Dithiocarbamates are also a known sulphur-containing lubricant additive and antiwear agent. Depending on their chemical properties and structures, dithiocarbamate additive promotes the economy of the fuel, boosts its load-carrying potential and reduces the possible wear and tear [207]. Tribological applications of several metal dithiocarbamate complexes have been investigated. Among the tested lubricant additives, molybdenum dialkyl dithiocarbamate was reported to be the most effective among the dithiocarbamate complexes based on the fuel economy, lubricant viscosity and driving cycle results [208]. Introduction of zinc dialkyldithio-phosphate to molybdenium dialky dithiocarbamate further improved the tribological properties of molybdenium dialky dithiocarbamate [209]. Shah et al. [210] investigated the comparative efficiencies of S-hydroxyethyl-*N*,*N*′-dibenzyldithiocarbamate (HE-BzDTC), *S*-(Di-*n*-butyl-borate)-ethyl-*N*,*N*′-dibenzyldithiocarbamate (DBB-EBzDTC), *S*-(Di-*n*-octyl-borate)-ethyl-*N*,*N*′-dibenzyldithiocarbamate (DOB-EBzDTC) and *S*-(Di-*n*-octyl-borate)-ethyl-*N*,*N*′-di-*n*-ethyldithiocarbamate (DOB-EEDTC) as lubricant additives. The performances of these dithiocarbamate compounds were compared with those without dithiocarbamate, and remarkable performance was observed compared to the additives without dithiocarbamates (as shown in Figure 12).

### 8.6. Application of Dithiocarbamate Compounds as Sensor

Chromogenic properties of dithiocarbamate anions are utilized in detecting both organic and inorganic pollutants. In some cases, dithiocarbamates are attached to other fluorescent moieties to sense pollutants even at a very low pollutant concentration [211]. Dithiocarbamate modified with gold was reported as sensor for divalent zinc through trimodal techniques. This sensor is significant because it can perfectly distinguish divalent cadmium from divalent zinc on the spot [212]. The ability of nickel dithiocarbamate-containing ortho isomer of sulforhodamine B to show a fluorescence increase when it reacts with nitrogen dioxide makes it a good sensor for nitrogen dioxide [213]. Apart from using dithiocarbamates alone as sensor, they have also been used to functionalize other materials used as sensor. For instance, the sensing of series of polyaromatic hydrocarbons has been made possible through the use of silver nanoparticles functionalized with dithiocarbamates [214]. Moreover, sensing of dithiocarbamate fungicide that is present in water and fruit juice was achieved through the use of silver nanoparticles functionalized with dopamine dithiocarbamate [215]. In addition to sensing metals, polyaromatic hydrocarbon, fungicides and gas, and dithiocarbamates have also found use in the sensing of anions. Bromide anion was detected when homoleptic cobalt(III) dithiocarbamate was used as sensor [216]. However, the lability of some of the dithiocarbamate complexes when they are in solution limited their use as sensor, but they become more applicable when they are attached to fluorescent moieties. This was adopted when organotin(IV) dithiocarbamate was added to antracene, which is a good fluorescent moiety to detect *O*-donor anions even when the concentrations of these anions were very low [211]. Other investigations involving the use of dithiocarbamate compounds as sensors are shown in Table 7.

## 9. Challenges Associated with the Utilization of Dithiocarbamates

Dithiocarbamates that possess aliphatic chains are vulnerable to acid hydrolysis and liberate CS_2_ under acidic or neutral conditions. In a very strong alkaline condition, the aliphatic dithiocarbamates degrade to give mixtures of sulphur-containing compounds such as sulfonates and disulphides [8]. Catalytic oxidation of thiols by dithiocarbamate compounds, leading to the inhibition of pro-apoptotic enzymes, has been reported [226]. Dithiocarbamate compounds also play significant roles in the disruption of the developmental stage of aquatic animals [227]. The product of metabolic degradation of dithiocarbamate (carbon disulphide) also causes notochord distortions in zebra fish [228]. Dithiocarbamate compounds have been found to possess biocidal and cytotoxic properties. Their cytotoxity was discovered to be related to their structures [229]. Disulfiram, thiram and mancozeb cause changes in the cell membrane and block glutamate from binding to the receptor, which results in toxic effects on the brain [230].

## 10. Conclusions and Future Perspectives

Dithiocarbamate may simply be the solution to the many environmental, medical, agricultural and industrial challenges based on their applications that have been highlighted in this review. The discussion presented herein is believed to inspire more studies and investigations into new applications of dithiocarbamate compounds. For future research, the use of dithiocarbamate complexes in medical imaging is still at the infant stage and it needs to be further explored. For instance, the possibility of using dithiocarbamate compounds to solve the problem of scattering, sensitivity and absorption in medical imaging should be investigated. Toxicity of the metal dithiocarbamate complexes should be thoroughly investigated prior to their use for these applications, so that any possible cytotoxic effect that could emanate from the introduction of dithiocarbamate into the ecosystem could be mitigated for the protection of aquatic and terrestrial lives. Moreover, the fate of the unused dithiocarbamate in the environment and their degradation mechanism through the use of photocatalysis and other removal methods should be studied. Furthermore, the effect of dithiocarbamate on the root exudates of common food crops such as maize and soy bean should be investigated so as to enhance food safety and productivity. While iron dithiocarbamate has been investigated for the removal of nitrogen oxides from air samples [231], this investigation needs to be carried out on other air pollutants. Finally, renewed efforts should be geared towards the synthesis of novel dithiocarbamate ligands and complexes.

## Figures and Tables

**Table 1:** Heavy metals remediation via dithiocarbamate.

Dithiocarbamate Compound Used	Heavy Metals Removed	Media/Samples Remediated	Amount Removed/Performance	Ref.
Iron-containing reduced graphene oxide modified with dithiocarbamate	Hg(II), Pb(II), Cd(II) and Cu(II)	wastewater	181.82, 147.06, 116.28 and 113.64 mg/g respectively	[23]
Dithiocarbamate-modified coal	Ni(II)	Aqueous solution	82.37 mg/g	[24]
Al(OH) -poly(acrylamide-dimethyldiallylammonium chloride)-graft-dithiocarbamate	Pb(II) and Cu(II)	Wastewater	17.777 mg/g for Cu and 586.699 mg/g for Pb	[25]
poly-sodium dithiocarbamate and poly-ammonium dithiocarbamate	Zn(II), Ni(II) and Cu(II)	Electroplating wastewater	226.76, 234.47 and 245.53 mg/g, for Zn, Ni and Cu respectively at pH 6 in 20 min	[26]
Heavy metal-dithiocarbamates (using sodium diethyldithiocarbamate)	Zn(II), Pb(II), Ni(II), Mn(II), Fe(II), Cu(II) and Cd(II) ions	Water sample	More than 90% removal	[14]
Sodium polyamidoamine-multi dithiocarbamate (using sodium diethyldithiocarbamate)	Divalent Zn, Cu, Cd and Pb	Soil sediments	Complete precipitation	[27]
sodium tetraethylenepentamine-multi dithiocarbamate	Divalent Cu, Cd and Pb	Soil samples	Near complete precipitation	[28]

**Table 2:** Determination of trace elements using dithiocarbamates.

Dithiocarbamate Compound Used	Metal(s) Determined	Method Used for the Determination	Limit of Detection	Ref.
pyrrolidine dithiocarbamate	Ni(II), Cr(VI), Co(II), and Hg(II)	liquid liquid micro-extraction	0.011–2.0 µg L^−1^	[36]
Ammonium 1-pyrrolidine dithiocarbamate and Diethylammonium diethyldithiocarbamate	Pb(II), Cu(II) and Cd(II)	Inductively coupled plasma-mass spectroscopy (ICP-MS)	0.13–1.18 pmol L^−1^	[37]
Ammonium pyrrolidine dithiocarbamate	As(III)	solid phase extraction(SPE)	0.01 μg L^−1^	[38]
Sodium diethyl dithiocarbamate	Cd(II) and Pb(II)	SPE/ FAAS	0.30 μg L ^−1^	[39]
Dithiocarbamate-functionalized magnetite composite	Hg(II)	Atomic absorption spectrometry with gold amalgamation	1.8 ng L^−1^	[40]
Pyrrolidine dithicarbamate	Pb(II), Bi(III), Pb(II), Hg(II), Au(III), Se(IV), As(III),Ni(II) and Co(II)	Thin-film microextraction	0.2–0.6 μg/L	[41]

**Table 3:** Application of dithiocarbamates in nanoparticle synthesis.

Dithiocarbamate Precursor Used	Nanoparticle(s) Obtained	Temp. Used	Particle Size and (Band Gap)	Ref.
Bis(N-ethylphenyldithiocarbamato) palladium(II)	Palladium sulphide	160, 200 and 240 °C resp.	2.01–2.50 nm, 4.00–4.86 nm and 2.53–4.12 nm (4.90–5.02 eV)	[57]
Bis(N,N-di(4-fluorobenzyl)dithiocarbamato-S,S′)M(II). (M = Cd)	Cadmium sulphide (CdS)	-	−(3.29 eV)	[58]
Cu (II) bis N-methyl-N-phenyl Dithiocarbamate	Copper sulphide (CuS and Cu_5_S_9_)	≥240 °C	34.7 ± 13.3 nm width size (1.85 eV)	[52]
Dithiocarbamate complexes with varied Ag/In/Ga/Zn ratios	Quinary Ag-In-Ga-Zn-S quantum dots	220 °C	2.0 ± 0.4 nm	[59]
Molybdenum dithiocarbamates	Molybdenium sulphide (MoS_2_)	-	40 nm	[60]
N-alkyldithiocarbamate copper(II) complexes with NaBH4	Copper sulphide (Cu_9_S_5_ and Cu_2_S)	180 °C	−(3.0 eV)	[61]
copper(ii) bis-(2,2′-(dithiocarboxyazanediyl)diacetic acid)	Copper sulphide (CuS)	90 °C	8 ± 1 nm	[62]
bis(diethyldithiocarbamato)disulfidothioxo tungsten(VI)	chromium-doped tungsten disulphide (WS_2_)	450 °C	-	[63]
tetrakis(N,N-diethyldithiocarbamato)molybdenum(IV)	Molybdenum sulphide (MoS_2_)	450 °C	flake thickness of ∼10 nm	[64]
[V_2_S_4_(nBu_2_dtc)_4_](dtc=dithiocarbamate)	Vanadium sulphide (VS_2_)	150 °C		[65]
Manganese diethyldithiocarbamate trihydrate	Manganese sulphide (MnS)	290 °C	(3.3 eV)	[66]
Tris-(piperidinedithiocarbamato) iron(III) and tris-(tetrahydroquinolinedithiocarbamato)iron(III)	Iron sulphide (Fe_0.975_S and Fe_3_S_4_ phases)	350–450 °C	(0.95–2.0 eV)	[67]
lead(II) complexes of morpholine dithiocarbamate	Lead sulphide (PbS)	160 °C	(13.86–36.06 nm)	[68]

**Table 4:** Scientific name of common dithiocarbamate pesticides and the organisms affected.

Dithiocarbamate Pesticides (Common Names)	Dithiocarbamate Pesticides (Scientific Names)	Classification	Organism(s) Affected	Ref.
Ferbam	Ferric dimethyldithiocarbamate	Fungicide	Drugs against gastrointestinal flukes, tapeworms, lungworms and roundworms in farm animals	[84]
mancozeb	Zinc;manganese(2+); N-[2-(sulfidocarbothioylamino)ethyl]carbamodithioate	Fungicide	Acts against over 400 micro-organisms that damage agricultural produce such as citrus, grapevine, tomato and potato	[85]
Carbaryl	1-naphthyl methyl carbamate	Insecticide	Acts against 100 species of destructive insects affecting pets, livestock, poultry, shade trees, ornamentals, nuts, lawns, forests, fruit and citrus	[86]
Maneb	Manganese-containing ethylene bis-dithiocarbamate	fungicide	To control the diseases of plants	[87,88]
metam-sodium	Methylisothiocyanate	Fungicide, nematocides and (herbicides)	To fumigate soil prior to planting so as to prevent soilborne diseases	[89]
Metiram	Zinc ammoniate ethlenebis(dithiocarbamate)-poly (ethylene disulphide)	Fungicide	Prevent plants(ornamentals, field, nuts, vegetables and fruits) by inhibiting the spores of the pathogens from germinating	[90,91]
Nabam	Ethylenebis[dithiocarbamic acid] disodium salt	Algaecide, bacteriacide and Fungicide	To prevent fungal diseases in tomato, apple and cotton and to eliminate algae from plant field	[92]
Thiram	Tetramethyl thiuram disulphide	Fungicide	It affects the mucous membrane and skin of microbes	[93]
Propineb	Polymeric zinc 1, 2-propylene bis(dithiocarbamate)	Fungicide	To treat fungal infections such as leaf blotch in apple and other crops.	[94]
Zineb	Zincethylenebis(dithiocarbamate)	Fungicide	To control the diseases of plants	[88]
Ziram	Zinc-dimethyl dithiocarbamate	Fungicide	To repel birds from flowers	[95]
Methiocarb	N-methylcarbamate	Insecticide	To repel birds from plants	[95]

**Table 5:** Different diseases treated with dithiocarbamate compounds.

Diseases/ Abnormality Treated	Brief Description of the Disease/ Abnormality	Dithiocarbamate Compound Used	Roles of Dithiocarbamate	Ref.
Influenza	Viral disease that affect the respiratory organs	Pyrrolidine dithiocarbamate	It acts against overproduction of reactive oxygen species and inhibit DNA fragmentation	[130]
Hyperglycemia	Too much of glucose in the bloodstream that may be as a result of diabetes mellitus	Allyldithiocar- bamates	Dithiocarbamates improved the sensitivity of insulin instead of the concentration of insulin leading to 18.2% glucose AUC (glucose area under the curve) in 15 days.	[131]
Tuberculosis	Bacterial infection that affect the lung	Several N,N-disubstituted and N-mono-dithiocarbamates	Treatment through the inhibition of carbonic anhydrase enzyme. These dithiocarbamate compounds were more effective as inhibitor than the clinically-approved sulfonamide.	[132]
Alzheimer disease	age-related neurodegenerative disorder	Several coumarin-dithiocarbamate	Treatment through the inhibition of acetylcholinesterase. They were able to reverse the cognative dysfunction	[133]
Dandruff	Fungal disease that affect the scalp leading to the shedding of dead skin cells.	Series of dithiocarbamates	Inhibition of β-class carbonic anhydrase of *Malassezia globosa*	[134]
Myasthenia gravis	An auto-immune disease causing the weakness of muscle	N,N-disubstituted dithiocarbamic acid	Treatment via inhibition of cholinesterase. They possessed better anticholinesterase properties more than Donepezil which is used for treating the disease.	[135]
SARS-CoV-2. (Still on clinical trial) NCT 04485130	Viral respiratory disease also known as coronavirus (COVID-19)	Disulfiram	Inhibition of viral replication and the anti-inflammatory activities leading to the treatment of the disease.	[9]
Alcoholism	Excessive and uncontrollable alcohol intake	Disulfiram	It inhibits acetaldehyde metabolism which is a product obtained from the breakdown of alcohol	[136,137]
Parkinson’s disease	Genetic disease associated with the loss of neuron	Pyrrolidine dithiocarbamate	It suppresses the level of glutamate	[138]
Male infertility	Inability to conceive children	Ziram	Reduction of the level of proteineous kinase by damaging the mitochondria ultrastructure thereby inhibiting human sperm motility.	[139]
Scorpionism	Painful condition as a result of scorpion sting	pyrrolidine dithiocarbamate	Inhibition of venom-induced thermal and mechanical hyperalgesia of *Tityus bahiensis.*	[140]

**Table 6:** Specific examples of antibacterial properties of dithiocarbamates against some bacterial strains.

Dithiocarbamate Compounds	Bacteria	Conc. of Isolation	Min. Inhibitory Conc. Range	Ref.
Phenyl dithiocarbamate mixed ligand metal complexes	*Escherichia coli, Proteus valgaris, Salmonella typhii, Shigella flexneri, Staphylococcus aureus, Bacillus subtilis, Streptococcus pneumonia, Psendomonas aeruginosa, Vibro chlolerae* and *Klebseilla pneumonia*	10 mg/mL	6–8 nm	[6]
sodium cyclohexyldithioc-arbamate and sodium phenyldithiocarbamate	*Salmonella typhi, Proteus mirabilis, Pseudomonas aeruginosa, Bacillus cereus* and *Bacillus subtilis*	15–30 mg/mL	(7.7–16.3 mm) and (8.5–19 mm) respectively	[162]
tris(ephedrinedithiocarbamate) complexes	*Pseudomona aeruginosa, Staphylococcus sciuri, Enterococcus caseofluvialis, Staphylococcus aureus, Enterobacter cloacae, Salmonella dublin, Klebsiella pneumonia* and *Escherichia coli*	25–100 μg/mL	14.6–126.5 μM	[163]
*N*-ethyl-*N*-phenyldithiocarbamate complexes	*Staphylococcus aureus, Salmonella typhi, Pseudomonas aureginosa* and *Escherichia coli*	100 μg/mL	-	[164]
Dibenzyldithiocarbamate	*Mycobacterium smegmatis Staphylococcus aureus, Pseudomonas aeruginosa and Escherichia coli*	0.5 mg/mL	64–1000 µg/mL	[165]
Rh(III)-morpholine-4-dithiocarbamate	*Salmonella typhai, Pseudomunas aeroginosa, Proteus mirabilis, Yersinia enterocolitica, Enterococcus faecalis Staphylococcus aureus*	50 ppm	5–28 mm	[166]
silver(I) dithiocarbamate triphenylphosphine	*Escherichia coli, Salmonella. typhimurium, Pseudomunas aeruginosa, Klebsiella pneumonia, Staphylococcus aureus*	1000 µg/mL	0.19–75.45 µM/mL	[159]
N-methyl-N-phenyl dithiocarbamate complexes of Cu(II), In(III) and Sb(III)	*Bacillus cereus, Enterococcus faecalis, Enterococcus gallinurium, Listeria monocytogenes, Listeria monocytogenes, Staphylococcus aureus, Escherichia coli, Klebsiella pneumonia, Salmonella enterica* and *Salmonella Typhimurium*	0.022–2.522 µg/mL	7.00–19.33 mm	[167]

**Table 7:** Application of dithiocarbamates in sensing.

Dithiocarbamate Compound	Substance Sensed	Detection Limits	Ref.
Chitosan dithiocarbamate	Divalent cadmium	63 nM.	[217]
Dithiocarbamate functionalized silver nanoparticles	Divalent cobalt	14 μM	[218]
ZnS quantum dots doped with glycine dithiocarbamate -functionalized Mn.	Trivalent cerium	2.29 × 10^−7^ mol.L^−1^	[219]
Nickel(II) dithiocarbamate complexes	Halide ions	-	[220]
Gold nanoparticles functionalized with Malonamide dithiocarbamate	Divalent mercury and copper	45 nM and 41 nM for Hg^2+^ and Cu^2+^ions respectively.	[221]
Silver nanoparticles functioalized with Cyclen dithiocarbamate	Paraquat and thiram pesticides	7.21 × 10^−6^ M and 2.81 × 10^−6^ M for paraquat and thiram respectively	[222]
Gold nanoparticles functionalized with *p*-amino salicylic acid dithiocarbamate	Trivalent iron	14.82 nM	[223]
Gold nanoparticles decorated with Ractopamine-dithiocarbamate	Pendimethalin herbicide	0.22 μM	[224]
Gold nanoparticles decorated with dithiocarbamate-p-tertbutylcalix[4]arene	Metsulfuron-methyl herbicide	1.9 × 10^−7^ M	[225]

## References

[1] Adeyemi J.O., Onwudiwe D.C. (2020). Chemistry and Some Biological Potential of Bismuth and Antimony Dithiocarbamate Complexes. Molecules.

[2] Cvek B., Dvorak Z. (2007). Targeting of nuclear factor-κB and proteasome by dithiocarbamate complexes with metals. Curr. Pharm. Des..

[3] Shinde S.D., Sakla A.P., Shankaraiah N. (2020). An insight into medicinal attributes of dithiocarbamates: Bird’s eye view. Bioorganic Chem..

[4] Chen N., Zhong X., Li P., Xu J. (2015). A Mild Radical Method for the Dimerzation of Dithiocarbamates. Eur. J. Org. Chem..

[5] Tan Y.S., Yeo C.I., Tiekink E.R.T., Heard P.J. (2021). Dithiocarbamate Complexes of Platinum Group Metals: Structural Aspects and Applications. Inorganics.

[6] Ejelonu B.C., Olagboye S.A., Oyeneyin O.E., Ebiesuwa O.A., Bada O.E. (2018). Synthesis, characterization and antimicrobial activities of sulfadiazine Schiff base and phenyl dithiocarbamate mixed ligand metal complexes. Open J. Appl. Sci..

[7] Kanchi S., Singh P., Bisetty K. (2014). Dithiocarbamates as hazardous remediation agent: A critical review on progress in environmental chemistry for inorganic species studies of 20th century. Arab. J. Chem..

[8] Szolar O.H.J. (2007). Environmental and pharmaceutical analysis of dithiocarbamates. Anal. Chim. Acta.

[9] Kaul L., Süss R., Zannettino A., Richter K. (2021). The revival of dithiocarbamates: From pesticides to innovative medical treatments. iScience.

[10] Ajiboye T.O., Oyewo O.A., Onwudiwe D.C. (2021). Photocatalytic removal of parabens and halogenated products in wastewater: A review. Environ. Chem. Lett..

[11] Ajiboye T.O., Oyewo O.A., Onwudiwe D.C. (2021). Adsorption and photocatalytic removal of Rhodamine B from wastewater using carbon-based materials. FlatChem.

[12] Ajiboye T.O., Oyewo O.A., Onwudiwe D.C. (2021). Simultaneous removal of organics and heavy metals from industrial wastewater: A review. Chemosphere.

[13] Ajiboye T.O., Kuvarega A.T., Onwudiwe D.C. (2020). Recent strategies for environmental remediation of organochlorine pesticides. Appl. Sci..

[14] Kane S., Lazo P., Ylli F., Stafilov T., Qarri F., Marku E. (2016). Separation of heavy metal from water samples—The study of the synthesis of complex compounds of heavy metal with dithiocarbamates. J. Environ. Sci. Health Part A.

[15] Nabipour H., Ghammamy S., Ashuri S., Aghbolaghc Z.S. (2010). Synthesis of a new dithiocarbamate compound and Study of Its biological properties. J. Org. Chem..

[16] Hogarth G., Rainford-Brent E.-J.C.R.C.R., Kabir S.E., Richards I., Wilton-Ely J.D.E.T., Zhang Q. (2009). Functionalised dithiocarbamate complexes: Synthesis and molecular structures of 2-diethylaminoethyl and 3-dimethylaminopropyl dithiocarbamate complexes [M{S2CN(CH2CH2NEt2)2}n] and [M{S2CN(CH2CH2CH2NMe2)2}n] (n = 2, M = Ni, Cu, Zn, Pd; n = 3, M = Co). Inorg. Chim. Acta.

[17] Tarique M., Aslam M. (2008). Bi and Trivalent transition metal complexes of dithiocarbamates derived from 2, 6-diacetyl pyridine. Orient. J. Chem..

[18] Abu-El-Halawa R., Zabin S.A. (2017). Removal efficiency of Pb, Cd, Cu and Zn from polluted water using dithiocarbamate ligands. J. Taibah Univ. Sci..

[19] Ayalew Z.M., Zhang X., Guo X., Ullah S., Leng S., Luo X., Ma N. (2020). Removal of Cu, Ni and Zn directly from acidic electroplating wastewater by Oligo-Ethyleneamine dithiocarbamate (OEDTC). Sep. Purif. Technol..

[20] Morita F., Nakakubo K., Yunoshita K., Endo M., Biswas F.B., Nishimura T., Mashio A.S., Hasegawa H., Taniguchi T., Maeda K. (2020). Dithiocarbamate-modified cellulose-based sorbents with high storage stability for selective removal of arsenite and hazardous heavy metals. RSC Adv..

[21] Li B., Guo J.Z., Liu J.L., Fang L., Lv J.Q., Lv K. (2020). Removal of aqueous-phase lead ions by dithiocarbamate-modified hydrochar. Sci. Total Environ..

[22] Zeng Q., Hu S., Zheng W., He Z., Zhou L., Huang Y. (2020). Spongy Crosslinked Branched Polyethylenimine-Grafted Dithiocarbamate: Highly Efficient Heavy Metal Ion-Adsorbing Material. J. Environ. Eng..

[23] Fu W., Huang Z. (2018). Magnetic dithiocarbamate functionalized reduced graphene oxide for the removal of Cu(II), Cd(II), Pb(II), and Hg(II) ions from aqueous solution: Synthesis, adsorption, and regeneration. Chemosphere.

[24] Liu Z., Han X., Ho C.H., Fan A. (2018). Adsorption of Ni^2+^ from aqueous solution by functionalized coal particles with dithiocarbamate. J. Hazard. Toxic Radioact. Waste.

[25] Liu Y., Qian P., Yu Y., Yu B., Wang Y., Ye S., Chen Y. (2018). Preparation and characterization of a novel hybrid chelating material for effective adsorption of Cu(II) and Pb(II). J. Environ. Sci..

[26] Chen H., Zhao Y., Yang Q., Yan Q. (2018). Preparation of poly-ammonium/sodium dithiocarbamate for the efficient removal of chelated heavy metal ions from aqueous environments. J. Environ. Chem. Eng..

[27] Deng T., Zhang B., Li F., Jin L. (2017). Sediment washing by EDTA and its reclamation by sodium polyamidoamine-multi dithiocarbamate. Chemosphere.

[28] Wang Y., Zhang B., Deng T., Li F. (2017). Reclamation of EDTA by sodium tetraethylenepentamine-multi dithiocarbamate after soil washing process with EDTA. Environ. Earth Sci..

[29] Srinivasan V., Subbaiyan M. (1989). Electroflotation Studies on Cu, Ni, Zn, and Cd with Ammonium Dodecyl Dithiocarbamate. Sep. Sci. Technol..

[30] Soylak M., Elci L. (1997). Preconcentration and separation of trace metal ions from sea water samples by sorption on amberlite XAD-16 after complexation with sodium diethyl dithiocarbamate. Int. J. Environ. Anal. Chem..

[31] Imyim A., Daorattanachai P., Unob F. (2013). Determination of Cadmium, Nickel, Lead, and Zinc in Fish Tissue by Flame and Graphite Furnace Atomic Absorption after Extraction with Pyrrolidine Dithiocarbamate and Activated Carbon. Anal. Lett..

[32] Cesur H. (2003). Determination of manganese, copper, cadmium and lead by FAAS after solid-phase extraction of their phenylpiperazine dithiocarbamate complexes on activated carbon. Turk. J. Chem..

[33] Lazaridou E., Kabir A., Furton K.G., Anthemidis A. (2020). A Novel Glass Fiber Coated with Sol-Gel Poly-Diphenylsiloxane Sorbent for the On-Line Determination of Toxic Metals Using Flow Injection Column Preconcentration Platform Coupled with Flame Atomic Absorption Spectrometry. Molecules.

[34] Smith R.M., Butt A.M., Thakur A. (1985). Determination of lead, mercury and cadmium by liquid chromatography using on-column derivatisation with dithiocarbamates. Analyst.

[35] Losev V.N., Parfenova V.V., Elsuf’ev E.V., Borodina E.V., Metelitsa S.I., Trofimchuk A.K. (2020). Separation and preconcentration followed by ICP-OES and ICP-MS determination of precious metals using silica gel chemically modified with dithiocarbamate groups. Sep. Sci. Technol..

[36] Laosuwan M., Mukdasai S., Srijaranai S. (2020). A simple in syringe low density solvent-dispersive liquid liquid microextraction for enrichment of some metal ions prior to their determination by high performance liquid chromatography in food samples. Molecules.

[37] Lu Y., Gao X., Chen C.T.A. (2019). Separation and determination of colloidal trace metals in seawater by cross-flow ultrafiltration, liquid-liquid extraction and ICP-MS. Mar. Chem..

[38] Santos L.B., de Oliveira D.M., de Souza A.O., Lemos V.A. (2019). A new method for the speciation of arsenic species in water, seafood and cigarette samples using an eggshell membrane. J. Iran. Chem. Soc..

[39] Kazantzi V., Drosaki E., Skok A., Vishnikin A.B., Anthemidis A. (2019). Evaluation of polypropylene and polyethylene as sorbent packing materials in on-line preconcentration columns for trace Pb(II)and Cd(II)determination by FAAS. Microchem. J..

[40] Tavares D.S., Vale C., Lopes C.B., Trindade T., Pereira E. (2019). Reliable quantification of mercury in natural waters using surface modified magnetite nanoparticles. Chemosphere.

[41] de la Calle I., Ruibal T., Lavilla I., Bendicho C. (2019). Direct immersion thin-film microextraction method based on the sorption of pyrrolidine dithiocarbamate metal chelates onto graphene membranes followed by total reflection X-ray fluorescence analysis. Spectrochim. Acta-Part B At. Spectrosc..

[42] Yeh C.F., Chyueh S.-D., Chen W.-S., Fang J.-D., Liu C.-Y. (1993). Application of dithiocarbamate resin-metal complexes as stationary phases in gas chromatography. J. Chromatogr. A.

[43] Bond A.M., Wallace G.G. (1984). Preparation of metal dithiocarbamate complexes for chromatographic separation and multi-element determinations. Anal. Chim. Acta.

[44] Aghbash K.O., Alamgholiloo H., Pesyan N.N., Khaksar S., Rostamnia S. (2021). Gold nanoparticle stabilized dithiocarbamate functionalized magnetite carbon as promise clean nanocatalyst for A3-coupling organic transformation. Mol. Catal..

[45] Pitchaimani P., Lo K.M., Elango K.P. (2015). Synthesis, crystal structures, luminescence properties and catalytic application of lanthanide(III) piperidine dithiocarbamate complexes. Polyhedron.

[46] Vale J.A., Faustino W.M., Menezes P.H., de Sá G.F. (2006). Eu(iii) dithiocarbamate complex and *N*-p-tolylsulfonylphenylalanine as a novel chiral catalyst for the asymmetric synthesis of cyanohydrins. Chem. Commun..

[47] Guan S., Zhong Z., Li J., Xu Y., Ding L., Huang Y., Liu L. (2021). Preparation of in-situ grown carbon nanotubes via dithiocarbamate in composites with excellent microstructure and mechanical performance. Compos. Sci. Technol..

[48] Uchiyama M., Satoh K., Kamigaito M. (2021). Stereospecific cationic RAFT polymerization of bulky vinyl ethers and stereoblock poly(vinyl alcohol) via mechanistic transformation to radical RAFT polymerization of vinyl acetate. Giant.

[49] Uchiyama M., Satoh K., Kamigaito M. (2015). Thioether-Mediated Degenerative Chain-Transfer Cationic Polymerization: A Simple Metal-Free System for Living Cationic Polymerization. Macromolecules.

[50] Uchiyama M., Satoh K., Kamigaito M. (2015). Cationic RAFT Polymerization Using ppm Concentrations of Organic Acid. Angew. Chem. Int. Ed..

[51] Huang Q., Liao H., Hu X., Cheng C. (2019). A cardanol-based surface-active dithiocarbamate and its application in emulsion polymerization. IOP Conf. Ser. Mater. Sci. Eng..

[52] Olatunde O.C., Onwudiwe D.C. (2021). Temperature Controlled Evolution of Pure Phase Cu9S5 Nanoparticles by Solvothermal Process. Front. Mater..

[53] Ajiboye T.O., Oyewo O.A., Onwudiwe D.C. (2021). The performance of bismuth-based compounds in photocatalytic applications. Surf. Interfaces.

[54] Srinivasan N. (2019). Fabrication and photocatalytic properties of Multi–Morphological CdS NSs prepared by the thermolysis of heterocyclic dithiocarbamate Cadmium(II) complexes as precursors. Dyes Pigments.

[55] Sarker J.C., Hogarth G. (2021). Dithiocarbamate Complexes as Single Source Precursors to Nanoscale Binary, Ternary and Quaternary Metal Sulfides. Chem. Rev..

[56] Mourdikoudis S., Liz-Marzán L.M. (2013). Oleylamine in Nanoparticle Synthesis. Chem. Mater..

[57] Paca A.M., Ajibade P.A. (2021). Bis-(*N*-ethylphenyldithiocarbamato)palladium(II) as molecular precursor for palladium sulfide nanoparticles. J. Mol. Struct..

[58] Eswari S., Selvaganapathi P., Thirumaran S., Ciattini S. (2021). Effect of solvent used for crystallization on structure: Synthesis and characterization of bis(*N*,*N*-di(4-fluorobenzyl)dithiocarbamato-S,S′)M(II) (M = Cd, Hg) and usage as precursor for CdS nanophotocatalyst. Polyhedron.

[59] Galiyeva P., Rinnert H., Balan L., Alem H., Medjahdi G., Uralbekov B., Schneider R. (2021). Single-source precursor synthesis of quinary AgInGaZnS QDs with tunable photoluminescence emission. Appl. Surf. Sci..

[60] Tanabe T., Osaki J., Miyajima M., Kitamura K., Oyama Y. (2021). Raman and TEM characterization of 2D layered MoS2 crystals grown on non-metal surfaces by friction-induced synthesis. Appl. Surf. Sci..

[61] Duran-García E.I., Martínez-Santana J., Torres-Gómez N., Vilchis-Nestor A.R., García-Orozco I. (2021). Copper sulfide nanoparticles produced by the reaction of *N*-alkyldithiocarbamatecopper(II) complexes with sodium borohydride. Mater. Chem. Phys..

[62] Mann P.B., McGregor I.J., Bourke S., Burkitt-Gray M., Fairclough S., Ma M.T., Hogarth G., Thanou M., Long N., Green M. (2019). An atom efficient, single-source precursor route to plasmonic CuS nanocrystals. Nanoscale Adv..

[63] Murtaza G., Venkateswaran S.P., Thomas A.G., O’Brien P., Lewis D.J. (2018). Chemical vapour deposition of chromium-doped tungsten disulphide thin films on glass and steel substrates from molecular precursors. J. Mater. Chem. C.

[64] Zeng N., Hopkinson D.G., Spencer B.F., McAdams S.G., Tedstone A.A., Haigh S.J., Lewis D.J. (2019). Direct synthesis of MoS_2_ or MoO_3_ via thermolysis of a dialkyl dithiocarbamato molybdenum(iv) complex. Chem. Commun..

[65] Fomenko I.S., Gushchin A.L., Nadolinny V.A., Efimov N.N., Laricheva Y.A., Sokolov M.N. (2018). Dinuclear Vanadium Sulfide Clusters: Synthesis, Redox Behavior, and Magnetic Properties. Eur. J. Inorg. Chem..

[66] Peng L., Shen S., Zhang Y., Xu H., Wang Q. (2012). Controllable synthesis of MnS nanocrystals from a single-source precursor. J. Colloid Interface Sci..

[67] Mlowe S., Lewis D.J., Malik M.A., Raftery J., Mubofu E.B., O’Brien P., Revaprasadu N. (2016). Heterocyclic dithiocarbamato-iron(iii) complexes: Single-source precursors for aerosol-assisted chemical vapour deposition (AACVD) of iron sulfide thin films. Dalton Trans..

[68] Dong Z., Jiang T., Xu B., Li Q., Zhong H., Yang Y. (2021). Selective flotation of galena using a novel collector S-benzyl-*N*-ethoxycarbonyl thiocarbamate: An experimental and theoretical investigation. J. Mol. Liq..

[69] Grainger R.S., Innocenti P. (2007). New applications of dithiocarbamates in organic synthesis. Heteroat. Chem..

[70] Ahmed S., Baker L.A., Grainger R.S., Innocenti P., Quevedo C.E. (2008). Thermal Elimination of Diethyldithiocarbamates and Application in the Synthesis of (±)-Ferrugine. J. Org. Chem..

[71] Jamir L., Sinha U.B., Nath J., Patel B.K. (2012). Environmentally Benign One-Pot Synthesis of Cyanamides from Dithiocarbamates Using I2 and H2O2. Synth. Commun..

[72] Yin B., Liu Z., Yi M., Zhang J. (2008). An efficient method for the synthesis of disubstituted thioureas via the reaction of *N*,*N*′-di-Boc-substituted thiourea with alkyl and aryl amines under mild conditions. Tetrahedron Lett..

[73] Halimehjani A.Z., Pourshojaei Y., Saidi M.R. (2009). Highly efficient and catalyst-free synthesis of unsymmetrical thioureas under solvent-free conditions. Tetrahedron Lett..

[74] Ziyaei-Halimehjani A., Marjani K., Ashouri A. (2012). A one-pot, three-component synthesis of thiazolidine-2-thiones. Tetrahedron Lett..

[75] Ziyaei Halimehjani A., Ranjbari M.A., Pasha Zanussi H. (2013). Synthesis of a new series of dithiocarbamate-linked peptidomimetics and their application in Ugi reactions. RSC Adv..

[76] Aryanasab F., Halimehjani A.Z., Saidi M.R. (2010). Dithiocarbamate as an efficient intermediate for the synthesis of 2-amino-1,3,4-thiadiazoles in water. Tetrahedron Lett..

[77] Raina-Fulton R. (2019). A Review of Methods for the Analysis of Orphan and Difficult Pesticides: Glyphosate, Glufosinate, Quaternary Ammonium and Phenoxy Acid Herbicides, and Dithiocarbamate and Phthalimide Fungicides. J. AOAC Int..

[78] Kaufman D.D. (1967). Degradation of carbamate herbicides in soil. J. Agric. Food Chem..

[79] Rogachev I., Kampel V., Gusis V., Cohen N., Gressel J., Warshawsky A. (1998). Synthesis, Properties, and Use of Copper-Chelating Amphiphilic Dithiocarbamates as Synergists of Oxidant-Generating Herbicides. Pestic. Biochem. Physiol..

[80] Abulnaja K.O., Harwood J.L. (1991). Thiocarbamate herbicides inhibit fatty acid elongation in a variety of monocotyledons. Phytochemistry.

[81] Wang Z., Yang L., Ye X., Huang C., Yang W., Zhang L., Wu Z., Fu F. (2020). Multicolor visual screening of total dithiocarbamate pesticides in foods based on sulfydryl-mediated growth of gold nanobipyramids. Anal. Chim. Acta.

[82] Eng G., Song X., Duong Q., Strickman D., Glass J., May L. (2003). Synthesis, structure characterization and insecticidal activity of some triorganotin dithiocarbamates. Appl. Organomet. Chem..

[83] Fan Z., Qin Y., Liu S., Xing R., Yu H., Chen X., Li K., Li R., Wang X., Li P. (2019). The bioactivity of new chitin oligosaccharide dithiocarbamate derivatives evaluated against nematode disease (*Meloidogyne incognita*). Carbohydr. Polym..

[84] Hussain A., Pu H., Hu B., Sun D.-W. (2021). Au@Ag-TGANPs based SERS for facile screening of thiabendazole and ferbam in liquid milk. Spectrochim. Acta Part A Mol. Biomol. Spectrosc..

[85] Runkle J., Flocks J., Economos J., Dunlop A.L. (2017). A systematic review of Mancozeb as a reproductive and developmental hazard. Environ. Int..

[86] Boran H., Altinok I., Capkin E. (2010). Histopathological changes induced by maneb and carbaryl on some tissues of rainbow trout, Oncorhynchus mykiss. Tissue Cell.

[87] Roede J.R., Jones D.P. (2014). Thiol-reactivity of the fungicide maneb. Redox Biol..

[88] Nash R.G., Beall M.L. (1980). Fate of maneb and zineb fungicides in microagroecosystem chambers. J. Agric. Food Chem..

[89] Triky-Dotan S., Ofek M., Austerweil M., Steiner B., Minz D., Katan J., Gamliel A. (2010). Microbial aspects of accelerated degradation of metam sodium in soil. Phytopathology.

[90] Chen M., Zhao Z., Lan X., Chen Y., Zhang L., Ji R., Wang L. (2015). Determination of carbendazim and metiram pesticides residues in reapeseed and peanut oils by fluorescence spectrophotometry. Measurement.

[91] Charles J.M., Tobia A., van Ravenzwaay B. (2000). Subchronic and Chronic Toxicological Investigations on Metiram: The Lack of a Carcinogenic Response in Rodents. Toxicol. Sci..

[92] Lin M.S., Wang J.S. (2004). Determination of an ethylene bisdithiocarbamate based pesticide (Nabam) by cobalt phthalocyanine modified carbon ink electrode. Electroanal. Int. J. Devoted Fundam. Pract. Asp. Electroanal..

[93] Zhang H., Mehmood K., Jiang X., Yao W., Iqbal M., Waqas M., Rehman M.U., Li A., Shen Y., Li J. (2018). Effect of tetramethyl thiuram disulfide (thiram) in relation to tibial dyschondroplasia in chickens. Environ. Sci. Pollut. Res..

[94] Zhou T., Zhao H., Huang L., Xi H., Zhou D., Cheng J. (2008). Efficacy of propineb for controlling leaf blotch caused by Marssonina coronaria and its effect on zinc content in apple leaves. Acta Phytophylacica Sin..

[95] Cummings J.L., Mason J.R., Otis D.L., Davis J.E., Ohashi T.J. (1994). Evaluation of methiocarb, ziram, and methyl anthranilate as bird repellents applied to dendrobium orchids. Wildl. Soc. Bull..

[96] Berry D.J., Torres Martin de Rosales R., Charoenphun P., Blower P.J. (2012). Dithiocarbamate complexes as radiopharmaceuticals for medical imaging. Mini Rev. Med. Chem..

[97] El-Aarag B.Y.A., Kasai T., Zahran M.A.H., Zakhary N.I., Shigehiro T., Sekhar S.C., Agwa H.S., Mizutani A., Murakami H., Kakuta H. (2014). In vitro anti-proliferative and anti-angiogenic activities of thalidomide dithiocarbamate analogs. Int. Immunopharmacol..

[98] Adokoh C.K. (2020). Therapeutic potential of dithiocarbamate supported gold compounds. RSC Adv..

[99] Morrison B.W., Doudican N.A., Patel K.R., Orlow S.J. (2010). Disulfiram induces copper-dependent stimulation of reactive oxygen species and activation of the extrinsic apoptotic pathway in melanoma. Melanoma Res..

[100] Elahabaadi E., Salarian A.A., Nassireslami E. (2021). Design, Synthesis, and Molecular Docking of Novel Hybrids of Coumarin-Dithiocarbamate Alpha-Glucosidase Inhibitors Targeting Type 2 Diabetes Mellitus. Polycycl. Aromat. Compd..

[101] Mollazadeh M., Mohammadi-Khanaposhtani M., Valizadeh Y., Zonouzi A., Faramarzi M.A., Kiani M., Biglar M., Larijani B., Hamedifar H., Mahdavi M. (2021). Novel Coumarin Containing Dithiocarbamate Derivatives as Potent alpha-Glucosidase Inhibitors for Management of Type 2 Diabetes. Med. Chem..

[102] Gao Y., Li L., Liu Y., Li W., Wang Z., Shou S., Chai Y. (2020). Effect of semaphorin-3A on the cellular stability of CD4^+^CD25^+^ regulatory T cells induced by lipopolysaccharide. Zhonghua Wei Zhong Bing Ji Jiu Yi Xue.

[103] Sağlık B.N., Osmaniye D., Çevik U.A., Levent S., Çavuşoğlu B.K., Büyükemir O., Nezir D., Karaduman A.B., Özkay Y., Koparal A.S. (2020). Synthesis, characterization and carbonic anhydrase I and II inhibitory evaluation of new sulfonamide derivatives bearing dithiocarbamate. Eur. J. Med. Chem..

[104] Aspatwar A., Parvathaneni N.K., Barker H., Anduran E., Supuran C.T., Dubois L., Lambin P., Parkkila S., Winum J.-Y. (2020). Design, synthesis, in vitro inhibition and toxicological evaluation of human carbonic anhydrases I, II and IX inhibitors in 5-nitroimidazole series. J. Enzym. Inhib. Med. Chem..

[105] Ge Y., Xu L.W., Liu Y., Sun L.Y., Gao H., Li J.Q., Yang K. (2019). Dithiocarbamate as a valuable scaffold for the inhibition of metallo-β-lactmases. Biomolecules.

[106] Asadi M., Ebrahimi M., Mohammadi-Khanaposhtani M., Azizian H., Sepehri S., Nadri H., Biglar M., Amanlou M., Larijani B., Mirzazadeh R. (2019). Design, Synthesis, Molecular Docking, and Cholinesterase Inhibitory Potential of Phthalimide-Dithiocarbamate Hybrids as New Agents for Treatment of Alzheimer’s Disease. Chem. Biodivers..

[107] Cihlar T., Fordyce M. (2016). Current status and prospects of HIV treatment. Curr. Opin. Virol..

[108] Takamune N., Misumi S., Shoji S. (2000). Cyclic Zinc-Dithiocarbamate-S,S′-Dioxide Blocks CXCR4-Mediated HIV-1 Infection1. Biochem. Biophys. Res. Commun..

[109] Pang H., Chen D., Cui Q.C., Ping Dou Q. (2007). Sodium diethyldithiocarbamate, an AIDS progression inhibitor and a copper-binding compound, has proteasome-inhibitory and apoptosis-inducing activities in cancer cells. Int. J. Mol. Med..

[110] Watanabe K., Kazakova I., Furniss M., Miller S.C. (1999). Dual activity of pyrrolidine dithiocarbamate on κB-dependent gene expression in U937 cells: I. Regulation by the phorbol ester TPA. Cell. Signal..

[111] Schreck R., Meier B., Männel D.N., Dröge W., Baeuerle P.A. (1992). Dithiocarbamates as potent inhibitors of nuclear factor kappa B activation in intact cells. J. Exp. Med..

[112] Ahlenstiel C.L., Suzuki K., Marks K., Symonds G.P., Kelleher A.D. (2015). Controlling HIV-1: Non-Coding RNA Gene Therapy Approaches to a Functional Cure. Front. Immunol..

[113] Lang J.-M., Trepo C., Kirstetter M., Herviou L., Retornaz G., Renoux G., Musset M., Touraine J.-L., Choutet P., Falkenrodt A. (1988). The Aids-Imuthiol French Study, G. Randomised, double-blind, placebo-controlled trial of ditiocarb sodium (’Imuthiol’) in human immunodeficiency virus infection. Lancet.

[114] Sunderman F.W.S. (1991). Therapeutic properties of sodium diethyldithiocarbamate: Its role as an inhibitor in the progression of AIDS. Ann. Clin. Lab. Sci..

[115] Hersh E.M., Brewton G., Abrams D., Bartlett J., Galpin J., Gill P., Gorter R., Gottlieb M., Jonikas J.J., Landesman S. (1991). Ditiocarb Sodium (Diethyldithiocarbamate) Therapy in Patients With Symptomatic HIV Infection and AIDS: A Randomized, Double-blind, Placebo-Controlled, Multicenter Study. JAMA.

[116] Bozdag M., Carta F., Vullo D., Akdemir A., Isik S., Lanzi C., Scozzafava A., Masini E., Supuran C.T. (2015). Synthesis of a new series of dithiocarbamates with effective human carbonic anhydrase inhibitory activity and antiglaucoma action. Bioorganic Med. Chem..

[117] Supuran C.T. (2021). Emerging role of carbonic anhydrase inhibitors. Clin. Sci..

[118] Francioli C., Wang X., Parapanov R., Abdelnour E., Lugrin J., Gronchi F., Perentes J., Eckert P., Ris H.-B., Piquilloud L. (2017). Pyrrolidine dithiocarbamate administered during ex-vivo lung perfusion promotes rehabilitation of injured donor rat lungs obtained after prolonged warm ischemia. PLoS ONE.

[119] Soerensen J., Pekcec A., Fuest C., Nickel A., Potschka H. (2009). Pyrrolidine dithiocarbamate protects the piriform cortex in the pilocarpine status epilepticus model. Epilepsy Res..

[120] Ebenezer P.J., Mariappan N., Elks C.M., Haque M., Soltani Z., Reisin E., Francis J. (2009). Effects of pyrrolidine dithiocarbamate on high-fat diet-induced metabolic and renal alterations in rats. Life Sci..

[121] Pal D.S., Mondal D.K., Datta R. (2015). Identification of Metal Dithiocarbamates as a Novel Class of Antileishmanial Agents. Antimicrob. Agents Chemother..

[122] Alvar J., Vélez I.D., Bern C., Herrero M., Desjeux P., Cano J., Jannin J., den Boer M., Team W.L.C. (2012). Leishmaniasis worldwide and global estimates of its incidence. PLoS ONE.

[123] Pandey K., Pun S.B., Pandey B.D. (2012). Relapse of kala-azar after use of multiple drugs: A case report and brief review of literature. Indian J. Med. Microbiol..

[124] Massai L., Messori L., Micale N., Schirmeister T., Maes L., Fregona D., Cinellu M.A., Gabbiani C. (2017). Gold compounds as cysteine protease inhibitors: Perspectives for pharmaceutical application as antiparasitic agents. BioMetals.

[125] Oliveira J.W.d.F., Rocha H.A.O., de Medeiros W.M.T.Q., Silva M.S. (2019). Application of Dithiocarbamates as Potential New Antitrypanosomatids-Drugs: Approach Chemistry, Functional and Biological. Molecules.

[126] Mezencev R., Galizzi M., Kutschy P., Docampo R. (2009). Trypanosoma cruzi: Antiproliferative effect of indole phytoalexins on intracellular amastigotes in vitro. Exp. Parasitol..

[127] Ochoa C., Perez E., Roland P., Suarez M., Ochoab E., Rodriguez H., Barrio A.G., Susana M., Nogal J.J., Martinez R.A. (1999). Synthesis and antiprotozoan properties of new 3,5-disubstituted-tetrahydro-2H-1,3,5-thiadiazine-2-thione derivatives. Arzneimittelforschung.

[128] Alam U., Asghar O., Azmi S., Malik R.A. (2014). General aspects of diabetes mellitus. Handb. Clin. Neurol..

[129] Chu G., Lei1 C., Qiu P., Hu Y., Meng X. (2017). Pyrrolidine dithiocarbamate alleviated anxiety in diabetic mice. Indian J. Pharm. Sci..

[130] Uchide N., Ohyama K., Bessho T., Yuan B., Yamakawa T. (2002). Effect of antioxidants on apoptosis induced by influenza virus infection: Inhibition of viral gene replication and transcription with pyrrolidine dithiocarbamate. Antivir. Res..

[131] Dighe S.U., Yadav V.D., Srivastava R., Mishra A., Gautam S., Srivastava A.K., Balaramnavar V.M., Saxena A.K., Batra S. (2014). Reinvestigations into synthesis of allyldithiocarbamates and their intramolecular cyclization: Synthesis and antihyperglycemic activity of 2-thioxothiazolidine-4-alkanoates. Tetrahedron.

[132] Maresca A., Carta F., Vullo D., Supuran C.T. (2013). Dithiocarbamates strongly inhibit the β-class carbonic anhydrases from Mycobacterium tuberculosis. J. Enzym. Inhib. Med. Chem..

[133] Jiang N., Huang Q., Liu J., Liang N., Li Q., Li Q., Xie S.-S. (2018). Design, synthesis and biological evaluation of new coumarin-dithiocarbamate hybrids as multifunctional agents for the treatment of Alzheimer’s disease. Eur. J. Med. Chem..

[134] Vullo D., Del Prete S., Nocentini A., Osman S.M., AlOthman Z., Capasso C., Bozdag M., Carta F., Gratteri P., Supuran C.T. (2017). Dithiocarbamates effectively inhibit the β-carbonic anhydrase from the dandruff-producing fungus Malassezia globosa. Bioorganic Med. Chem..

[135] Mohsen U., Kaplancikli Z., Özkay Y., Yurttaş L. (2015). Synthesis and evaluation of anti-acetylcholinesterase activity of some benzothiazole based new piperazine-dithiocarbamate derivatives. Drug Res..

[136] Brewer C. (1993). Recent developments in disulfiram treatment. Alcohol Alcohol..

[137] O’Farrell T.J., Allen J.P., Litten R.Z. (1995). Disulfiram (antabuse) contracts in treatment of alcoholism. NIDA Res. Monogr..

[138] Abdelkader N.F., Arafa N.M., Attia A.S., Ain-Shoka A.A., Abdallah D.M. (2017). Pyrrolidine dithiocarbamate ameliorates rotenone-induced Parkinson’s disease in rats. Bull. Fac. Pharm. Cairo Univ..

[139] Wen Z., Lei Z., Tian E., Wang Y., Zhong Y., Ge R.S. (2021). Inhibition of human sperm motility and capacitation by ziram is mediated by decreasing tyrosine protein kinase. Ecotoxicol. Environ. Saf..

[140] Ferraz C.R., Manchope M.F., Andrade K.C., Saraiva-Santos T., Franciosi A., Zaninelli T.H., Bagatim-Souza J., Borghi S.M., Cândido D.M., Knysak I. (2021). Peripheral mechanisms involved in Tityus bahiensis venom-induced pain. Toxicon.

[141] Vane J.R., Botting R.M. (2003). The mechanism of action of aspirin. Thromb. Res..

[142] Dinarello C.A. (2010). Anti-inflammatory Agents: Present and Future. Cell.

[143] Song Z., Zhou Y., Zhang W., Zhan L., Yu Y., Chen Y., Jia W., Liu Z., Qian J., Zhang Y. (2019). Base promoted synthesis of novel indole-dithiocarbamate compounds as potential anti-inflammatory therapeutic agents for treatment of acute lung injury. Eur. J. Med. Chem..

[144] Lamkanfi M. (2011). Emerging inflammasome effector mechanisms. Nat. Rev. Immunol..

[145] Li C.-W., Chen Z.-W., Wu X.-L., Ning Z.-X., Su Z.-Q., Li Y.-C., Su Z.-R., Lai X.-P. (2015). A Standardized Traditional Chinese Medicine Preparation Named Yejuhua Capsule Ameliorates Lipopolysaccharide-Induced Acute Lung Injury in Mice via Downregulating Toll-Like Receptor 4/Nuclear Factor-kB. Evid.-Based Complementary Altern. Med..

[146] Topping R.J., Jones M.M. (1988). Optimal dithiocarbamate structure for immunomodulator action. Med. Hypotheses.

[147] Cuzzocrea S., Chatterjee P.K., Mazzon E., Dugo L., Serraino I., Britti D., Mazzullo G., Caputi A.P., Thiemermann C. (2020). Pyrrolidine dithiocarbamate attenuates the development of acute and chronic inflammation. Br. J. Pharmacol..

[148] Bhalla Y., Gupta V.K., Jaitak V. (2013). Anticancer activity of essential oils: A review. J. Sci. Food Agric..

[149] Reddy L., Odhav B., Bhoola K.D. (2003). Natural products for cancer prevention: A global perspective. Pharmacol. Ther..

[150] Fu D.J., Li J.H., Yang J.J., Li P., Zhang Y.B., Liu S., Li Z.R., Zhang S.Y. (2019). Discovery of novel chalcone-dithiocarbamates as ROS-mediated apoptosis inducers by inhibiting catalase. Bioorganic Chem..

[151] Milacic V., Chen D., Giovagnini L., Diez A., Fregona D., Dou Q.P. (2008). Pyrrolidine dithiocarbamate-zinc(II) and -copper(II) complexes induce apoptosis in tumor cells by inhibiting the proteasomal activity. Toxicol. Appl. Pharmacol..

[152] Lawal M.M., Lawal I.A., Klink M.J., Tolufashe G.F., Ndagi U., Kumalo H.M. (2020). Density functional theory study of gold(III)-dithiocarbamate complexes with characteristic anticancer potentials. J. Inorg. Biochem..

[153] Omar A.M.M.E., AboulWafa O.M., El-Shoukrofy M.S., Amr M.E. (2020). Benzoxazole derivatives as new generation of anti-breast cancer agents. Bioorganic Chem..

[154] Bakthavatsalam S., Wiangnak P., George D.J., Zhang T., Franz K.J. (2020). Dithiocarbamate prodrugs activated by prostate specific antigen to target prostate cancer. Bioorganic Med. Chem. Lett..

[155] Wang H., Wei J., Jiang H., Zhang Y., Jiang C., Ma X. (2021). Design, synthesis and pharmacological evaluation of three novel dehydroabietyl piperazine dithiocarbamate ruthenium (II) polypyridyl complexes as potential antitumor agents: DNA damage, cell cycle arrest and apoptosis induction. Molecules.

[156] Syed Annuar S.N., Kamaludin N.F., Awang N., Chan K.M. (2021). Cellular Basis of Organotin(IV) Derivatives as Anticancer Metallodrugs: A Review. Front. Chem..

[157] Adeyemi J.O., Onwudiwe D.C. (2020). The mechanisms of action involving dithiocarbamate complexes in biological systems. Inorg. Chim. Acta.

[158] Manoussakis G., Bolos C., Ecateriniadou L., Sarris C. (1987). Synthesis, characterization and anti-bacterial studies of mixed-ligand complexes of dithiocarbamato—thiocyanato and iron(III), nickel(II), copper(II) and zinc(II). Eur. J. Med. Chem..

[159] Oladipo S.D., Tolufashe G.F., Mocktar C., Omondi B. (2021). Ag(I) symmetrical *N*,*N*′-diarylformamidine dithiocarbamate PPh3 complexes: Synthesis, structural characterization, quantum chemical calculations and in vitro biological studies. Inorg. Chim. Acta.

[160] Chen Q.M., Li Z., Tian G.X., Chen Y., Wu X.H. (2021). 1,2,3-triazole-dithiocarbamate-naphthalimides: Synthesis, characterization, and biological evaluation. J. Chem. Res..

[161] Mohammed M.H., Leelon A.A. (2021). Synthesis, characterization of isatin dithiocarbamate derivatives with expected biological activities. Int. J. Drug Deliv. Technol..

[162] Ndukwe G.I., Nzeneri J.U., Abayeh O.J. (2018). Antibacterial assay of two synthesized dithiocarbamate ligands. Am. J. Chem. Appl..

[163] Ariza-Roldán A.O., López-Cardoso E.M., Rosas-Valdez M.E., Roman-Bravo P.P., Vargas-Pineda D.G., Cea-Olivares R., Acevedo-Quiroz M., Razo-Hernández R.S., Alvarez-Fitz P., Jancik V. (2017). Synthesis, characterization, antimicrobial and theoretical studies of the first main group tris(ephedrinedithiocarbamate) complexes of As(III), Sb(III), Bi(III), Ga(III) and In(III). Polyhedron.

[164] Onwudiwe D.C., Ekennia A.C. (2017). Synthesis, characterization, thermal, antimicrobial and antioxidant studies of some transition metal dithiocarbamates. Res. Chem. Intermed..

[165] Pastrana-Dávila A., Amaya-Flórez A., Aranaga C., Ellena J., Macías M., Flórez-López E., D’Vries R.F. (2021). Synthesis, characterization, and antibacterial activity of dibenzildithiocarbamate derivates and Ni(II)–Cu(II) coordination compounds. J. Mol. Struct..

[166] Mansouri G., Ghobadi M., Notash B. (2021). Synthesis, spectroscopic, structural, DFT and antibacterial studies of cyclometalated rhodium(III) complex based on morpholinedithiocarbamate ligand. Inorg. Chem. Commun..

[167] Ajiboye T.O., Oluwarinde B.O., Montso P.K., Ateba C.N., Onwudiwe D.C. (2021). Antimicrobial activities of Cu(II), In(III), and Sb(III) complexes of *N*-methyl-*N*–phenyl dithiocarbamate complexes. Results Chem..

[168] Menezes D.C., Vieira F.T., de Lima G.M., Wardell J.L., Cortés M.E., Ferreira M.P., Soares M.A., Vilas Boas A. (2008). The in vitro antifungal activity of some dithiocarbamate organotin(IV) compounds on Candida albicans—A model for biological interaction of organotin complexes. Appl. Organomet. Chem..

[169] Qin Y., Liu S., Xing R., Yu H., Li K., Meng X., Li R., Li P. (2012). Synthesis and characterization of dithiocarbamate chitosan derivatives with enhanced antifungal activity. Carbohydr. Polym..

[170] Badawy M.E.I., Rabea E.I., Bautista-Baños S., Romanazzi G., Jiménez-Aparicio A. (2016). Chapter 7-Chitosan and Its Derivatives as Active Ingredients Against Plant Pests and Diseases. Chitosan in the Preservation of Agricultural Commodities.

[171] Ferreira I.P., de Lima G.M., Paniago E.B., Takahashi J.A., Pinheiro C.B. (2014). Synthesis, characterization and antifungal activity of new dithiocarbamate-based complexes of Ni(II), Pd(II) and Pt(II). Inorg. Chim. Acta.

[172] Fargasova A., Reinprecht L., Kizlink J. (1997). Efficiency of organotin dithiocarbamate derivatives against wood destroying fungi. Biologia.

[173] Duran A., Valero N., Mosquera J., Fuenmayor E., Alvarez-Mon M. (2017). Gefitinib and pyrrolidine dithiocarbamate decrease viral replication and cytokine production in dengue virus infected human monocyte cultures. Life Sci..

[174] Lin L., Qin Y., Wu H., Chen Y., Wu S., Si X., Wang H., Wang T., Zhong X., Zhai X. (2015). Pyrrolidine dithiocarbamate inhibits enterovirus 71 replication by down-regulating ubiquitin–proteasome system. Virus Res..

[175] Mathur A., Mallia M.B., Subramanian S., Banerjee S., Kothari K., Dhotare B., Sarma H.D., Venkatesh M. (2005). 99mTcN complexes of tert-butyl dithiocarbamate and methoxyisobutyl dithiocarbamate as myocardial and brain imaging agents. Nucl. Med. Commun..

[176] Torres Martin de Rosales R., Tavaré R., Paul R.L., Jauregui-Osoro M., Protti A., Glaria A., Varma G., Szanda I., Blower P.J. (2011). Synthesis of 64CuII–bis (dithiocarbamatebisphosphonate) and its conjugation with superparamagnetic iron oxide nanoparticles: In vivo evaluation as dual-modality PET–MRI agent. Angew. Chem..

[177] Zhang J., Guo H., Zhang S., Lin Y., Wang X. (2008). Synthesis and biodistribution of a novel 99mTcN complex of ciprofloxacin dithiocarbamate as a potential agent for infection imaging. Bioorganic Med. Chem. Lett..

[178] Lin X., Jin Z., Ren J., Pang Y., Zhang W., Huo J., Wang X., Zhang J., Zhang Y. (2012). Synthesis and Biodistribution of a New 99mTc-oxo Complex with Deoxyglucose Dithiocarbamate for Tumor Imaging. Chem. Biol. Drug Des..

[179] Hait S., Valentín J.L., Jiménez A.G., Ortega P.B., Ghosh A.K., Stöckelhuber K.W., Wießner S., Heinrich G., Das A. (2020). Poly(acrylonitrile-co-butadiene) as polymeric crosslinking accelerator for sulphur network formation. Heliyon.

[180] Oenslager G. (1933). Organic Accelerators. Ind. Eng. Chem..

[181] Wang Y., Lü Y., Hu S., Hu T., Wen S., Liu L. (2019). Application of Lanthanum Diethyldithiocarbamate as Rubber Accelerator Used in Nitrile Butadiene Rubber. J. Chin. Rare Earth Soc..

[182] Palaty S., Joseph R. (2004). Synergism of Xanthate/Dithiocarbamate Accelerator in Carbon Black Filled NR Compounds. Iran. Polym. J..

[183] Zou Y., He J., Tang Z., Zhu L., Luo Y., Liu F. (2015). Effect of multifunctional samarium lysine dithiocarbamate on curing properties, static and dynamic mechanical properties of SBR/silica composites. RSC Adv..

[184] Alam M.N., Mandal S.K., Roy K., Debnath S.C. (2014). Safe amine based zinc dithiocarbamates for the vulcanization of carbon black reinforced natural rubber. J. Appl. Polym. Sci..

[185] Guo A.J., Pan H.H., Zheng W.L., Jiao S.H., Wang F., Jin Z.Z., Liu H., Chen K., Wang Z.X. (2019). Synthesis of dispersed molybdenum disulfide nano-catalysts and their performance in the hydrogenation of simulated oil slurry. J. Fuel Chem. Technol..

[186] Yang S., Liu L., Jia Z., Jia D., Luo Y. (2011). Study on the curing properties of SBR/La-GDTC/SiO_2_ composites. J. Rare Earths.

[187] Pudovik A.N., Khairullin V.K., Il’yasov A.V., Vasyanina M.A., Aleksandrova I.A., Ismayev I.E., Ovcharov V.I. (1988). Mechanism of action of phosphorylated dithiocarbamates on the vulcanization of rubbers. Polym. Sci. USSR.

[188] Nieuwenhuizen P.J., Ehlers A.W., Haasnoot J.G., Janse S.R., Reedijk J., Baerends E.J. (1999). The Mechanism of Zinc(II)-Dithiocarbamate-Accelerated Vulcanization Uncovered; Theoretical and Experimental Evidence. J. Am. Chem. Soc..

[189] Liu S., Dong Y., Xie L., Liu G., Zhong H., Zeng H. (2021). Uncovering the hydrophobic mechanism of a novel dithiocarbamate-hydroxamate surfactant towards galena. Chem. Eng. Sci..

[190] Liu B., Wang X., Du H., Liu J., Zheng S., Zhang Y., Miller J.D. (2016). The surface features of lead activation in amyl xanthate flotation of quartz. Int. J. Miner. Processing.

[191] Feng Q.-C., Wen S.-M., Zhao W.-J., Cao Q.-B., Lü C. (2016). A novel method for improving cerussite sulfidization. Int. J. Miner. Metall. Mater..

[192] Elizondo-Álvarez M.A., Uribe-Salas A., Nava-Alonso F. (2020). Flotation studies of galena (PbS), cerussite (PbCO3) and anglesite (PbSO4) with hydroxamic acids as collectors. Miner. Eng..

[193] Huang X., Jia Y., Cao Z., Wang S., Ma X., Zhong H. (2019). Investigation of the interfacial adsorption mechanisms of 2-hydroxyethyl dibutyldithiocarbamate surfactant on galena and sphalerite. Colloids Surf. A Physicochem. Eng. Asp..

[194] Ngobeni W.A., Hangone G. (2013). The effect of using sodium di-methyl-dithiocarbamate as a co-collector with xanthates in the froth flotation of pentlandite containing ore from Nkomati mine in South Africa. Miner. Eng..

[195] Qi J., Dong Y., Liu S., Liu G. (2021). A selective flotation of cassiterite with a dithiocarbamate-hydroxamate molecule and its adsorption mechanism. Appl. Surf. Sci..

[196] Matveeva T.N., Gromova N.K., Lantsova L.B. (2020). Analysis of Complexing and Adsorption Properties of Dithiocarbamates Based on Cyclic and Aliphatic Amines for Gold Ore Flotation. J. Min. Sci..

[197] Matveeva T.N., Chanturia V.A., Gromova N.K., Lantsova L.B. (2019). New compositions of agents for fine gold recovery from tailings. Gorn. Zhurnal.

[198] Wang J.B., Zhao F., Yang X.L., Han W.Y., Long K., Zhou Y.R. (2014). Marine Environmental Risk Assessment Method for Active Substances Used in Antifouling Systems on Ships in China. Advanced Materials Research.

[199] Nagata S., Zhou X., Okamura H. (2008). Antagonistic and Synergistic Effects of Antifouling Chemicals in Mixture. Encyclopedia of Ecology, Five-Volume Set.

[200] Parviz M., Darwish N., Alam M.T., Parker S.G., Ciampi S., Gooding J.J. (2014). Investigation of the Antifouling Properties of Phenyl Phosphorylcholine-Based Modified Gold Surfaces. Electroanalysis.

[201] Narayanan T.S.N., Subbaiyan M. (1994). Effect of dithiocarbamates on the phase constituents, alkaline stability, and wet adhesion of phosphate coatings. Met. Finish..

[202] Narayanan T.S.N.S., Subbaiyan M. (1993). Effect of surfactants on the porosity and corrosion resistance of zinc-phosphated steel. Met. Finish..

[203] Belzile N., Chen Y.-W., Cai M.-F., Li Y. (2004). A review on pyrrhotite oxidation. J. Geochem. Explor..

[204] Shu X., Dang Z., Zhang Q., Yi X., Lu G., Guo C., Yang C. (2013). Passivation of metal-sulfide tailings by covalent coating. Miner. Eng..

[205] Zhou Y., Qu J. (2017). Ionic Liquids as Lubricant Additives: A Review. ACS Appl. Mater. Interfaces.

[206] Kenbeek D., Buenemann T., Rieffe H. (2000). Review of Organic Friction Modifiers-Contribution to Fuel Efficiency.

[207] Rastogi R.B., Maurya J.L., Jaiswal V., Tiwary D. (2012). Lanthanum dithiocarbamates as potential extreme pressure lubrication additives. Int. J. Ind. Chem..

[208] Yamamoto K., Hiramatsu T., Hanamura R., Moriizumi Y., Heiden S. (2019). The Study of Friction Modifiers to Improve Fuel Economy for WLTP with Low and Ultra-Low Viscosity Engine Oil.

[209] Wang Y., Yue W., Kang J., Zhu L., Fu Z., Wang C. (2019). Effect of Surface Nanocrystallization Pretreatment on the Tribological Properties of Plasma Nitrided AISI 316 L Stainless Steel Under Boundary Lubrication. J. Tribol..

[210] Shah F.U., Glavatskih S., Antzutkin O.N. (2012). Novel Alkylborate–Dithiocarbamate Lubricant Additives: Synthesis and Tribophysical Characterization. Tribol. Lett..

[211] Fuentes-Martínez J.P., Toledo-Martínez I., Román-Bravo P., Garcia y García P., Godoy-Alcántar C., López-Cardoso M., Morales-Rojas H. (2009). Diorganotin(IV) dithiocarbamate complexes as chromogenic sensors of anion binding. Polyhedron.

[212] Gao R., Li D., Zheng S., Gu H., Deng W. (2021). Colorimetric/fluorescent/Raman trimodal sensing of zinc ions with complexation-mediated Au nanorod. Talanta.

[213] Yan Y., Krishnakumar S., Yu H., Ramishetti S., Deng L.-W., Wang S., Huang L., Huang D. (2013). Nickel(II) Dithiocarbamate Complexes Containing Sulforhodamine B as Fluorescent Probes for Selective Detection of Nitrogen Dioxide. J. Am. Chem. Soc..

[214] Guerrini L., Garcia-Ramos J.V., Domingo C., Sanchez-Cortes S. (2009). Sensing Polycyclic Aromatic Hydrocarbons with Dithiocarbamate-Functionalized Ag Nanoparticles by Surface-Enhanced Raman Scattering. Anal. Chem..

[215] Rohit J.V., Solanki J.N., Kailasa S.K. (2014). Surface modification of silver nanoparticles with dopamine dithiocarbamate for selective colorimetric sensing of mancozeb in environmental samples. Sens. Actuators B Chem..

[216] Gurumoorthy G., Rani P.J., Thirumaran S., Ciattini S. (2017). Cobalt(III) dithiocarbamates for anion sensing and preparation of cobalt sulfide and cobalt-iron sulfide nanoparticles: Photocatalytic degradation of dyes with as-prepared nanoparticles. Inorg. Chim. Acta.

[217] Mehta V.N., Basu H., Singhal R.K., Kailasa S.K. (2015). Simple and sensitive colorimetric sensing of Cd^2+^ ion using chitosan dithiocarbamate functionalized gold nanoparticles as a probe. Sens. Actuators B Chem..

[218] Mehta V.N., Mungara A.K., Kailasa S.K. (2013). Dopamine dithiocarbamate functionalized silver nanoparticles as colorimetric sensors for the detection of cobalt ion. Anal. Methods.

[219] Rofouei M.K., Tajarrod N., Masteri-Farahani M., Zadmard R. (2015). A New Fluorescence Sensor for Cerium (III) Ion Using Glycine Dithiocarbamate Capped Manganese Doped ZnS Quantum Dots. J. Fluoresc..

[220] Sathiyaraj E., Gurumoorthy G., Thirumaran S. (2015). Nickel(ii) dithiocarbamate complexes containing the pyrrole moiety for sensing anions and synthesis of nickel sulfide and nickel oxide nanoparticles. New J. Chem..

[221] Mehta V.N., Kailasa S.K. (2015). Malonamide dithiocarbamate functionalized gold nanoparticles for colorimetric sensing of Cu^2+^ and Hg^2+^ ions. RSC Adv..

[222] Rohit J.V., Kailasa S.K. (2014). Cyclen dithiocarbamate-functionalized silver nanoparticles as a probe for colorimetric sensing of thiram and paraquat pesticides via host–guest chemistry. J. Nanoparticle Res..

[223] Mehta V.N., Kailasa S.K., Wu H.-F. (2014). Sensitive and selective colorimetric sensing of Fe^3+^ ion by using p-amino salicylic acid dithiocarbamate functionalized gold nanoparticles. New J. Chem..

[224] Rohit J.V., Kailasa S.K. (2017). Simple and selective detection of pendimethalin herbicide in water and food samples based on the aggregation of ractopamine-dithiocarbamate functionalized gold nanoparticles. Sens. Actuators B Chem..

[225] Rohit J.V., Singhal R.K., Kailasa S.K. (2016). Dithiocarbamate-calix[4]arene functionalized gold nanoparticles as a selective and sensitive colorimetric probe for assay of metsulfuron-methyl herbicide via non-covalent interactions. Sens. Actuators B Chem..

[226] Tonkin E.G., Valentine H.L., Zimmerman L.J., Valentine W.M. (2003). Parenteral *N*,*N*-diethyldithiocarbamate produces segmental demyelination in the rat that is not dependent on cysteine carbamylation. Toxicol. Appl. Pharmacol..

[227] Tilton F., La Du J.K., Tanguay R.L. (2008). Sulfhydryl systems are a critical factor in the zebrafish developmental toxicity of the dithiocarbamate sodium metam (NaM). Aquat. Toxicol..

[228] Tilton F., La Du J.K., Vue M., Alzarban N., Tanguay R.L. (2006). Dithiocarbamates have a common toxic effect on zebrafish body axis formation. Toxicol. Appl. Pharmacol..

[229] Van Leeuwen C.J., Maas-Diepeveen J.L., Niebeek G., Vergouw W.H.A., Griffioen P.S., Luijken M.W. (1985). Aquatic toxicological aspects of dithiocarbamates and related compounds. I. Short-term toxicity tests. Aquat. Toxicol..

[230] Lushchak V.I., Matviishyn T.M., Husak V.V., Storey J.M., Storey K.B. (2018). Pesticide toxicity: A mechanistic approach. EXCLI J..

[231] Fujii S., Yoshimura T. (2000). A new trend in iron–dithiocarbamate complexes: As an endogenous NO trapping agent. Coord. Chem. Rev..

